# Supplementary material for: Fault-mediated magma propagation and triggered seismicity revealed by the 2022 São Jorge Azores unrest
Source: Nat Commun. 2026 Apr 23;17:3531. doi: 10.1038/s41467-026-71668-6 (PMC13106810; doi:10.1038/s41467-026-71668-6)
Supplement: Supplementary file 1 — Supplementary information [file 41467_2026_71668_MOESM1_ESM.pdf]

Supplementary Information

to

Fault-mediated magma propagation and triggered seismicity revealed by the 2022 São Jorge Azores unrest

by Stephen P. Hicks, Pablo J. Gonzalez, *et al*.

Supplementary Figures

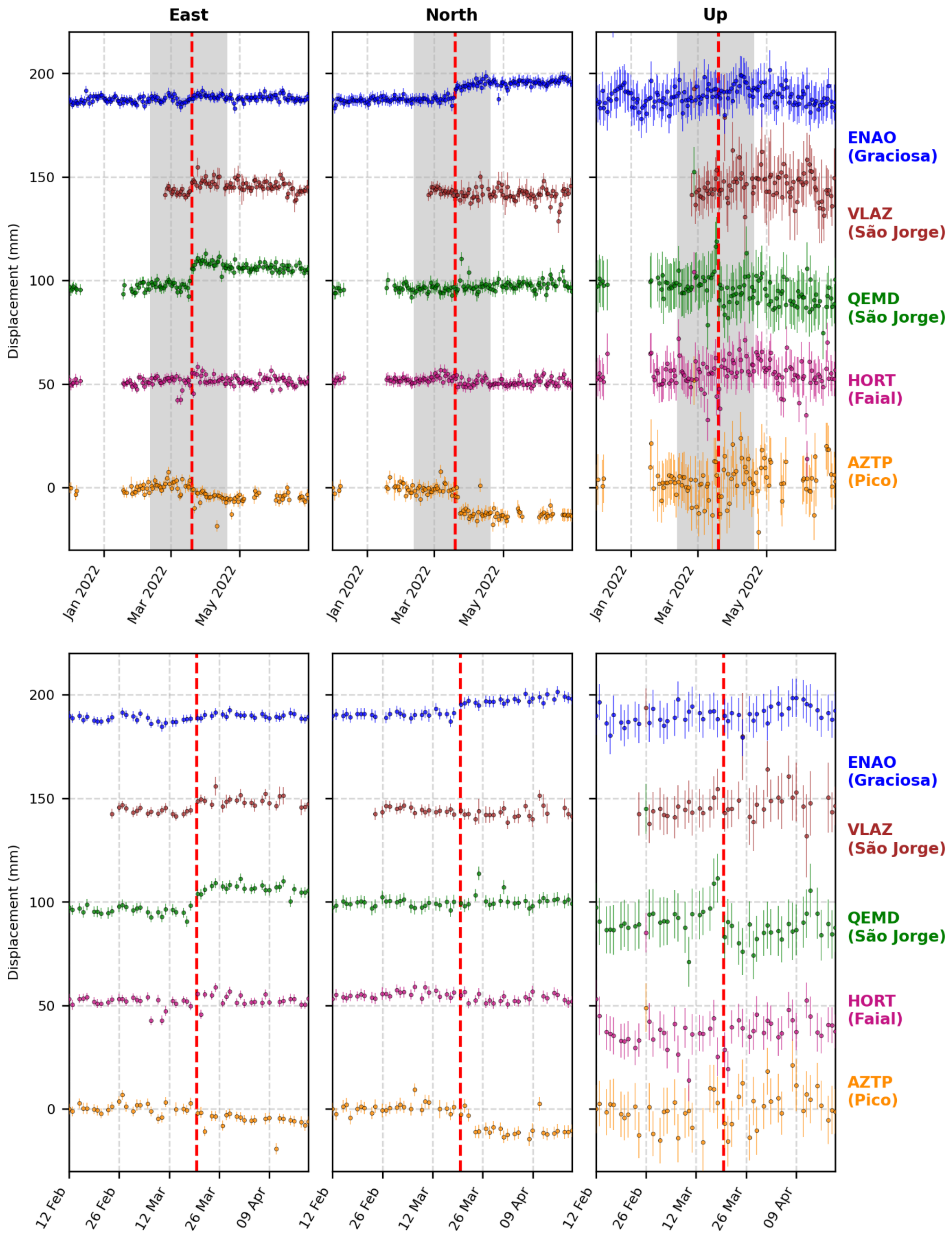

Fig. S1: East (left), north (middle), and vertical (right) daily GNSS displacements, with associated errors, from stations across the Central Group of the Azores (Fig. 1b). The bottom row shows a zoom-in view as given by the grey shaded region in the top row. Stations are ordered from north (top) to south (bottom). Displacements are referenced to a global reference frame, with secular

trends removed. The vertical red dashed line shows the onset of the seismic crisis (19 March 2022).

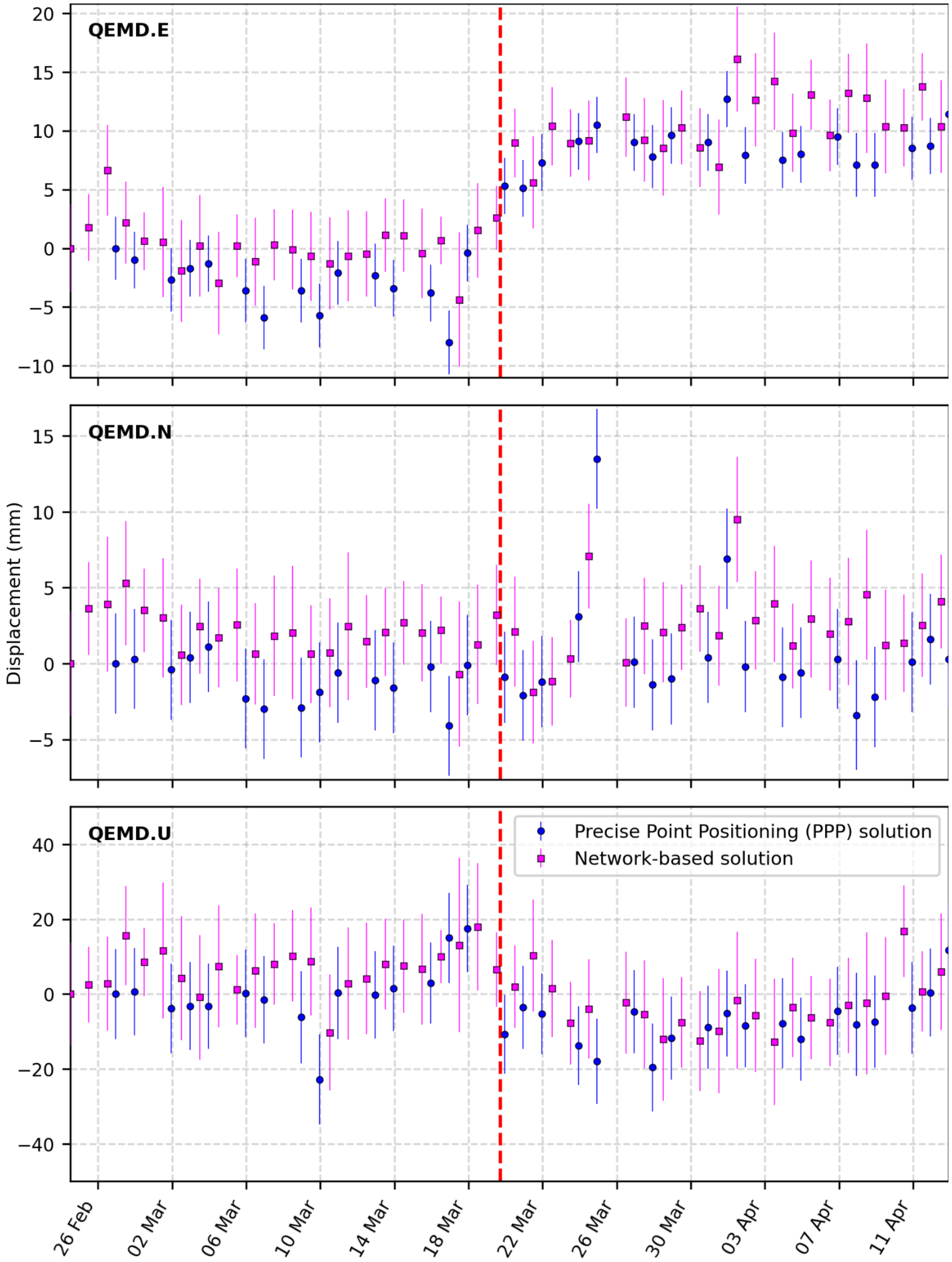

Fig. S2: Comparison between precise point position (PPP; as shown elsewhere in this paper) and network-based daily GNSS solutions for station QEMD.

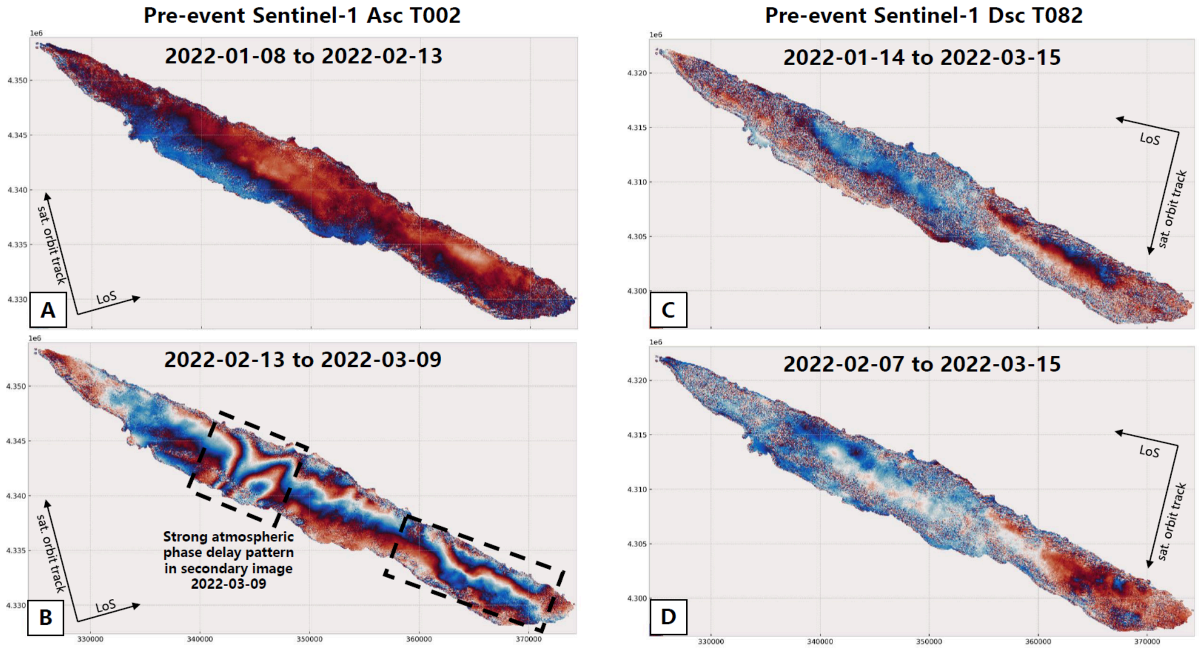

Fig. S3: Wrapped phase (C-band) ascending (A and B panels) and descending (C and D panels) Sentinel-1A interferograms from before the onset of seismicity showing that there were no InSAR detectable displacements (> 1 cm) before 15 March 2022 in São Jorge. Note that Panel B shows a strong phase pattern in the area of maximum co-diking displacements; however, pair-wise comparisons with multiple interferograms indicate that this phase delay pattern is likely due to an atmospheric phase delay affecting the 9 March 2022 radar image, as demonstrated by Panel D, which spans a similar period.

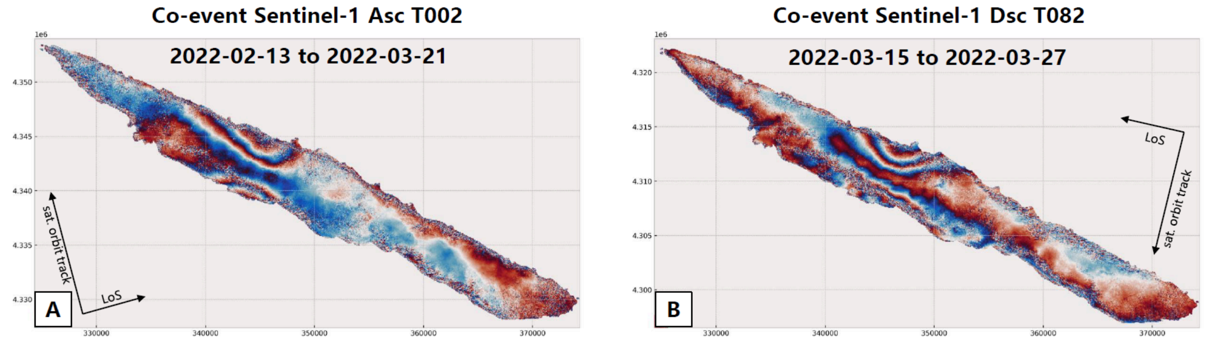

Fig. S4: Wrapped phase (C-band) ascending (A; same as Fig. 1c) and descending (B) Sentinel-1A co-onset of seismicity interferograms, showing that both ascending and descending pairs detected displacements with similar amplitude, indicating that most of the deformation occurred between 15 March 2022 and 21 March 2022 in São Jorge.

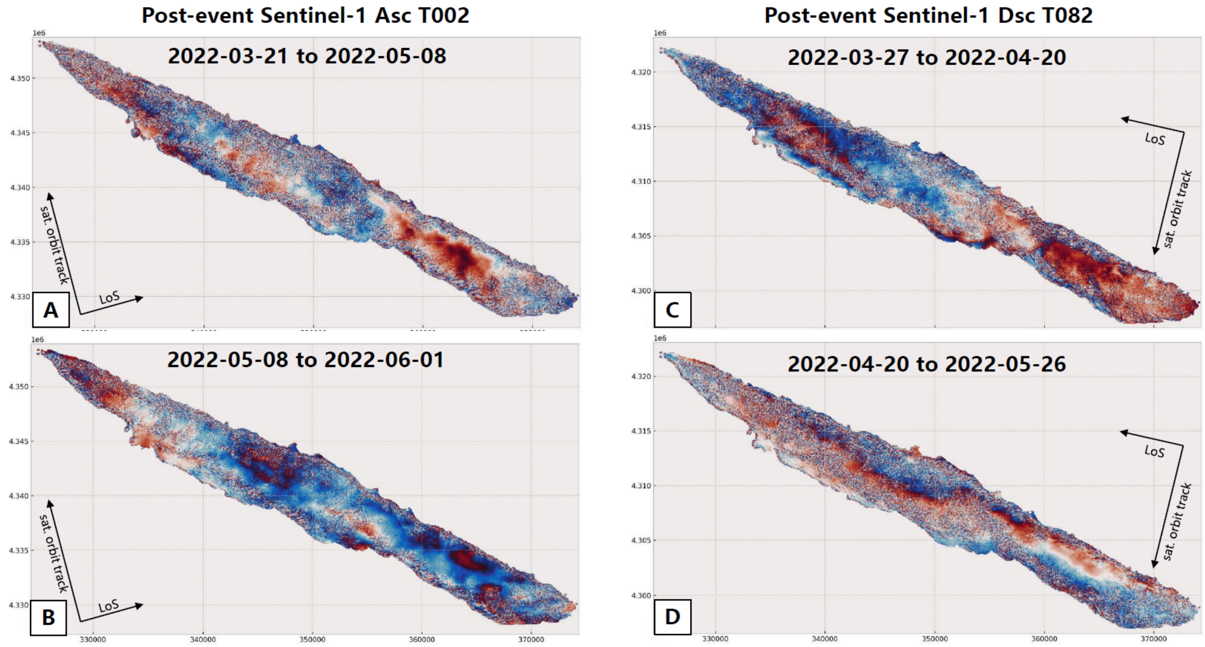

Fig. S5: Wrapped phase (C-band) ascending (A and B panels) and descending (C and D panels) Sentinel-1A post-onset of seismicity interferograms, showing that there were no InSAR detectable displacements (> 1 cm) after 21 March 2022 in São Jorge.

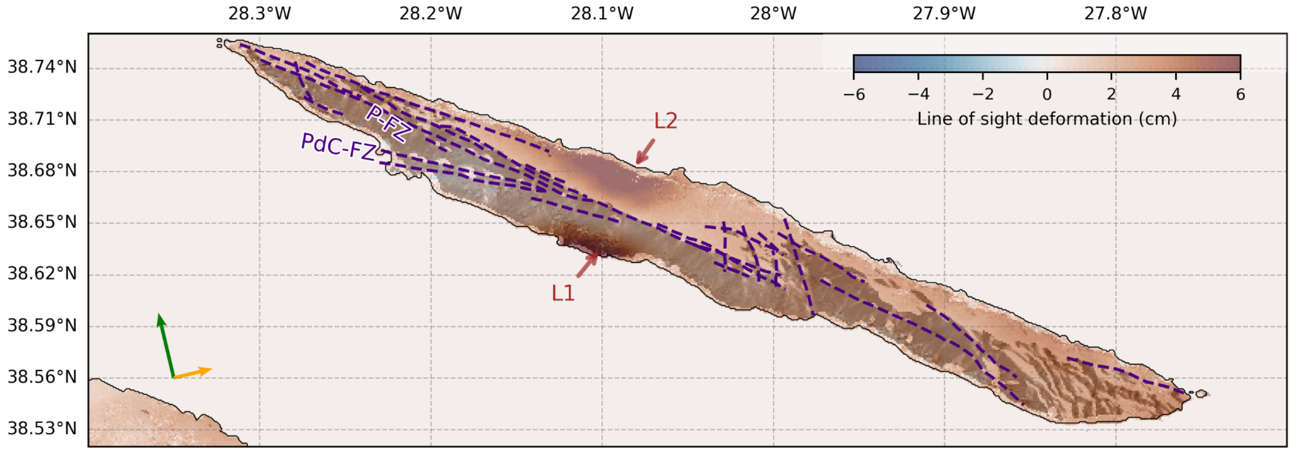

Fig. S6: Same as Fig. 1C, instead showing the unwrapped ascending interferogram from the Sentinel-1A satellite spanning 2022-02-13 - 2022-03-21. The green and orange arrows show the satellite azimuth and look direction, respectively. The brown annotations indicate the two uplift lobes in the interferogram. Indigo-coloured dashed lines show mapped faults and volcanic alignments on São Jorge[1,2].

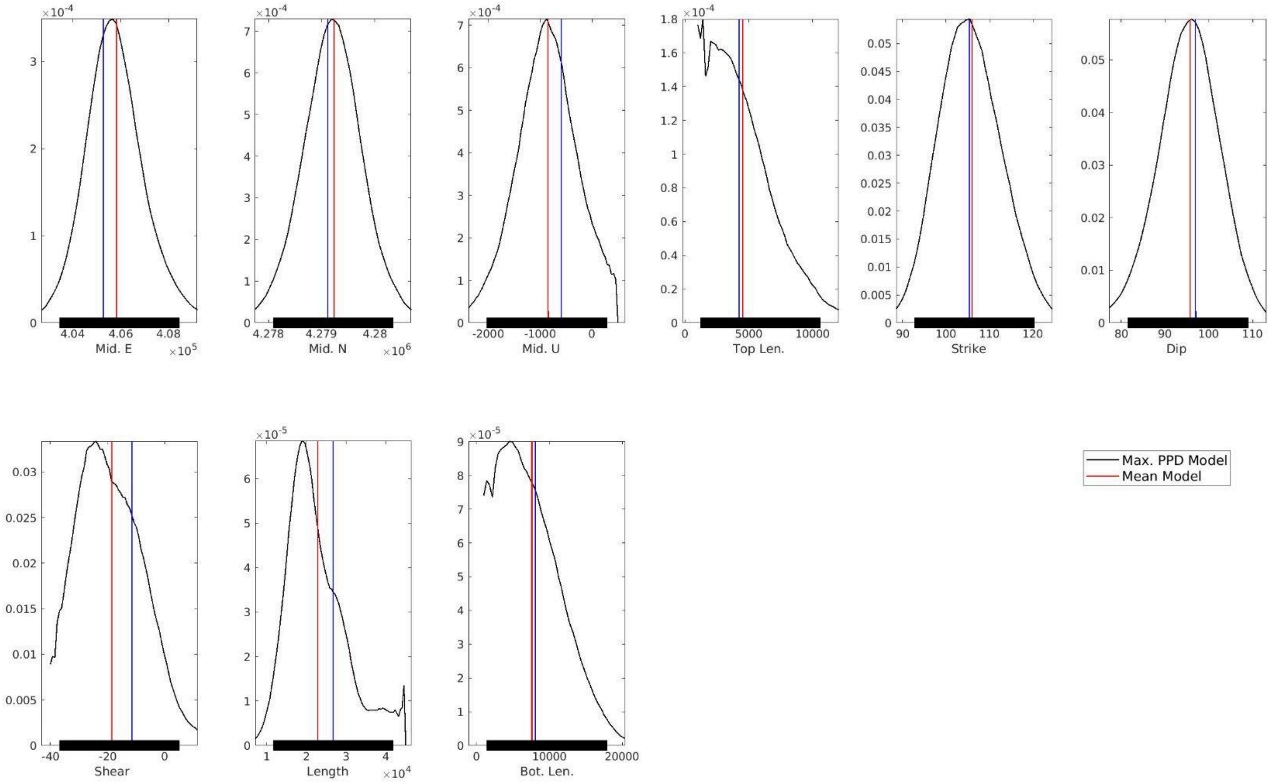

Fig. S7. Marginal probability density functions of the inverted parameters of the GNSS vectors and ascending and descending InSAR displacement maps for the quadrangular diking model. The horizontal black bars represent the confidence limits from the inverted model solutions.

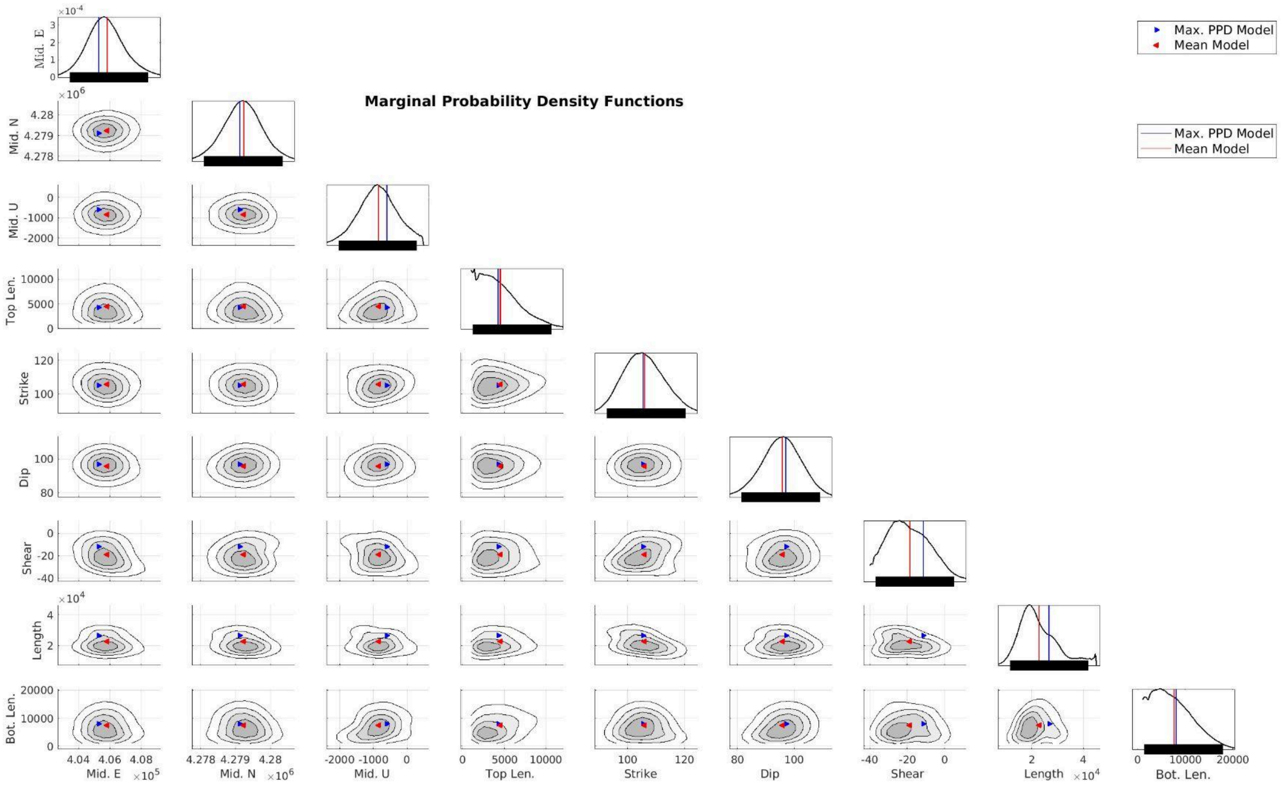

Fig. S8. 2D Marginal probability density functions of the inverted parameters of the GNSS vectors and ascending and descending InSAR displacement maps for the quadrangular diking model. Circular 2D distributions indicate that the inversion samples the parameter space well, with no strong trade-off correlations affecting the inverted model parameters. The horizontal black bars represent the confidence limits from the inverted model solutions. See Table S1 for a thorough description of the geometric parameters “Mid. E” = Easting, “Mid. N” = Northing, “Mid. U” = Top Depth; “Top Len.” = Top Length; “Bot. Len.” = Bottom Length; “Nb. Iter.” = Number of iterations.

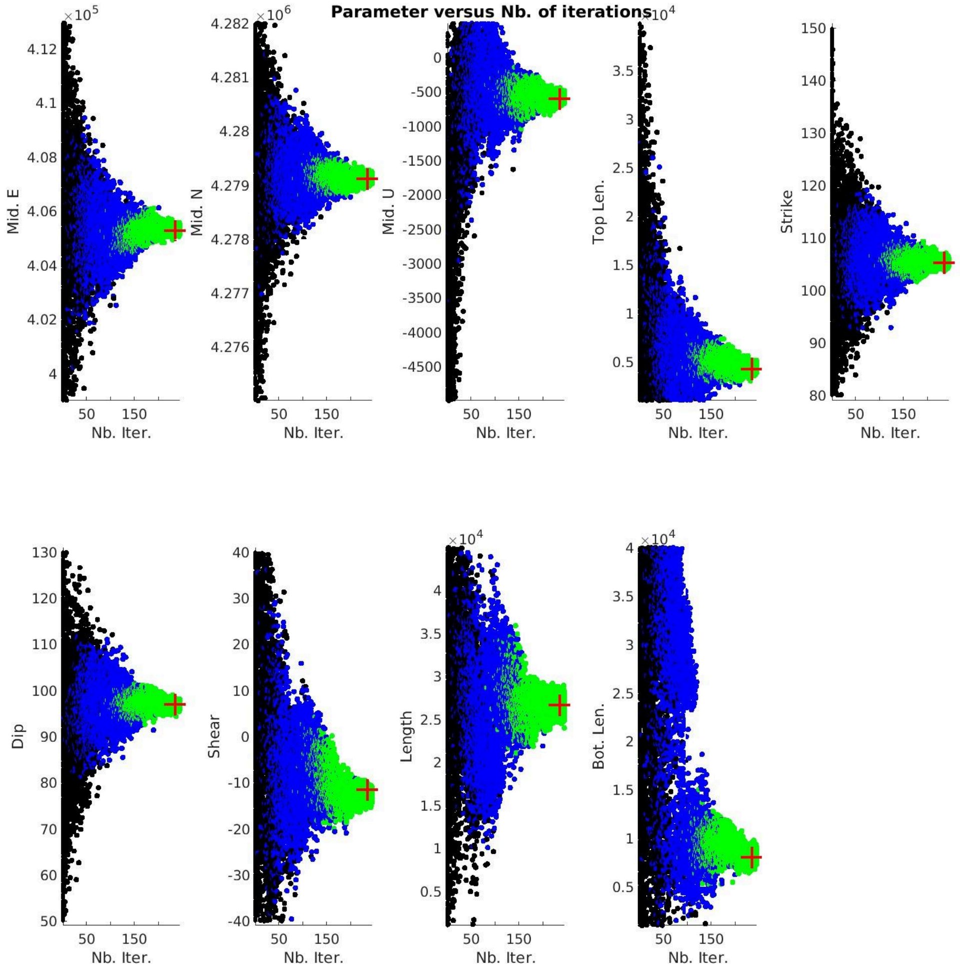

Fig. S9. Convergence of the non-linear inversion for the overall cost function for individual geometric parameters. The inversion terminates after 250 iterations, when the model “cost” dropped below 750 and was stable for approximately the last 100 iterations, indicating that the solution had stabilised. Blue points are those <20% of the initial cost function value; green points are those <2% of the initial cost value. The red “+” indicates the final accepted model. See Table S1 for a thorough description of the geometric parameters “Mid. E” = Easting, “Mid. N” = Northing, “Mid. U” = Top Depth; “Top Len.” = Top Length; “Bot. Len.” = Bottom Length; “Nb. Iter.” = Number of iterations.

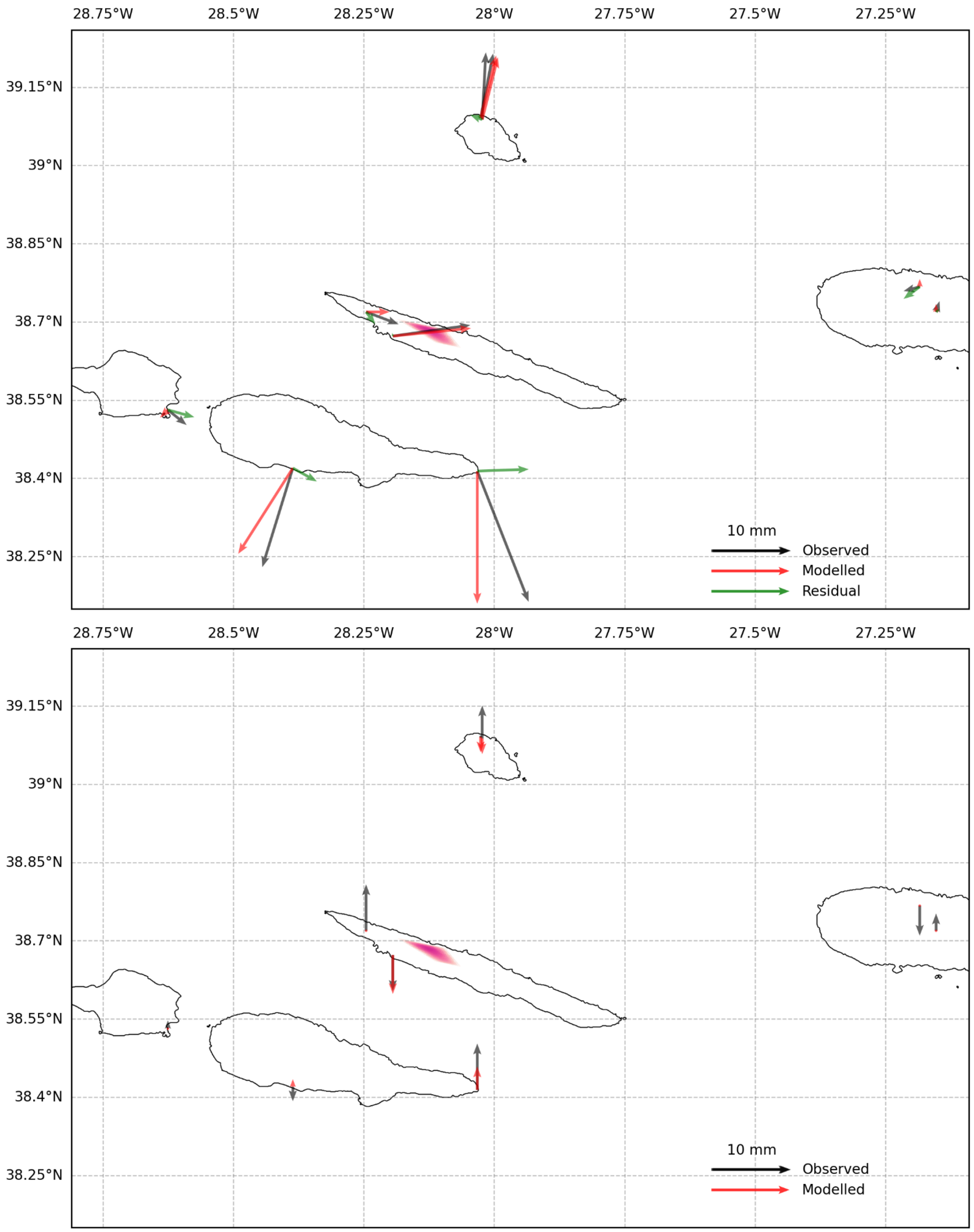

Fig S10: Observed, predicted, and residual GNSS horizontal (top) and vertical (bottom) displacement vectors from our joint BEM dike intrusion model. Our preferred dike intrusion model is shown in purple.

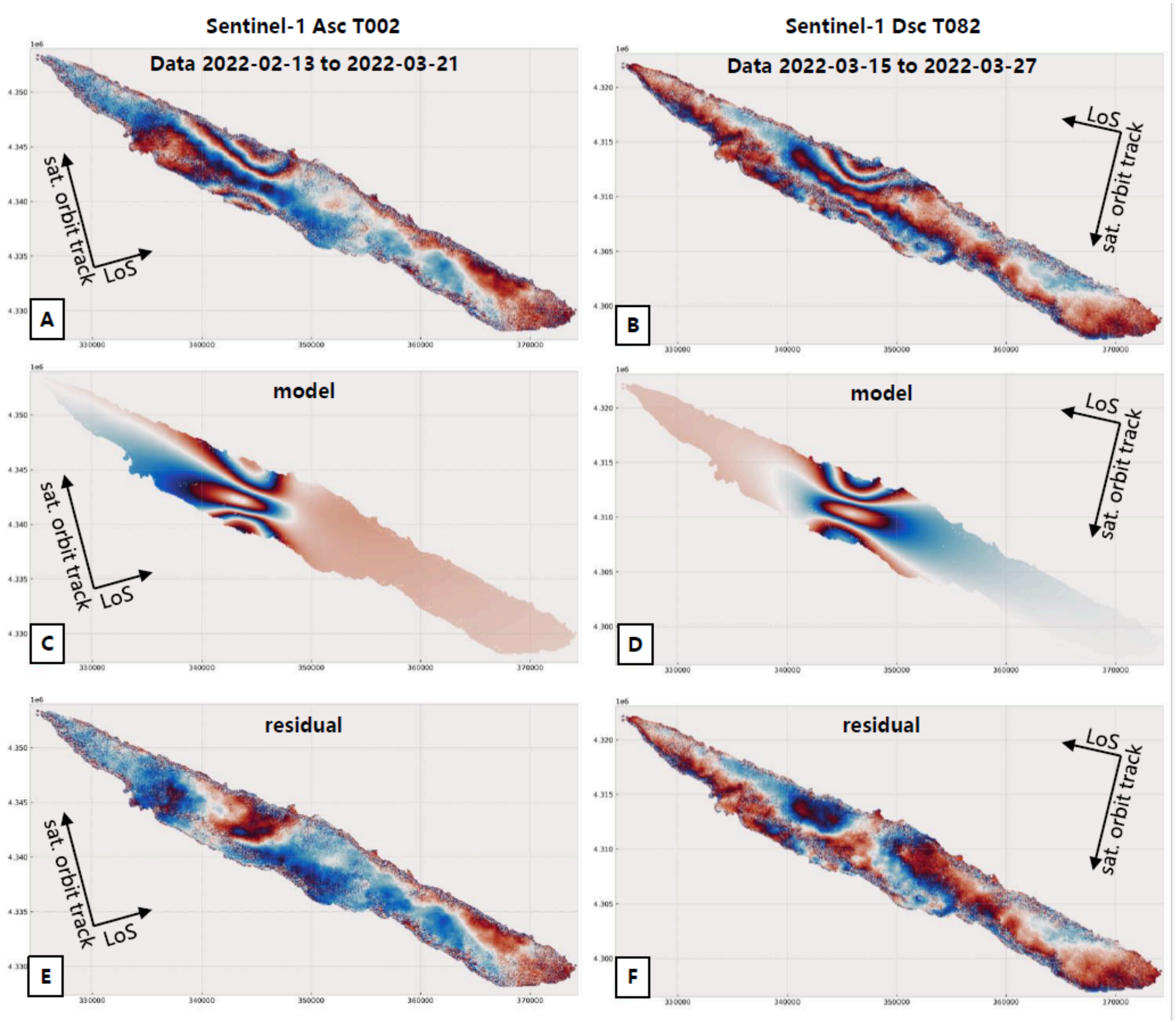

Fig S11: Ascending and descending (re-wrapped phase) InSAR displacement (A and B panels), model prediction (C and D panels) and residual (E and F panels) maps for São Jorge Island from our joint GNSS and InSAR inversion BEM dike intrusion model, including realistic topography-bathymetry.

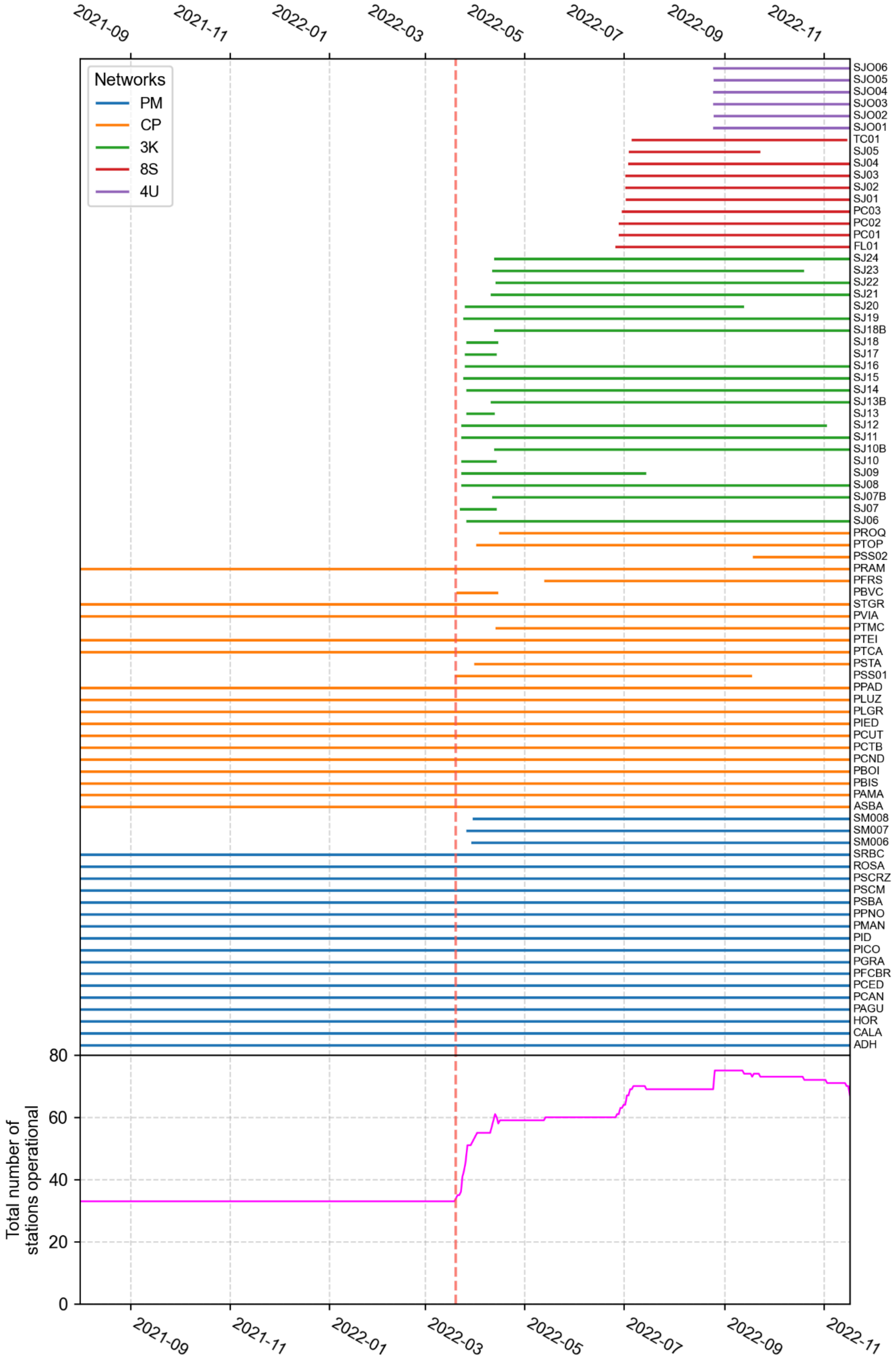

Fig. S12: Timeline showing the operational periods of seismic stations. Each line corresponds to an individual station, whose code is labelled on the right. Line colours correspond to networks. The bottom panel shows the total number of stations operational at a given time.

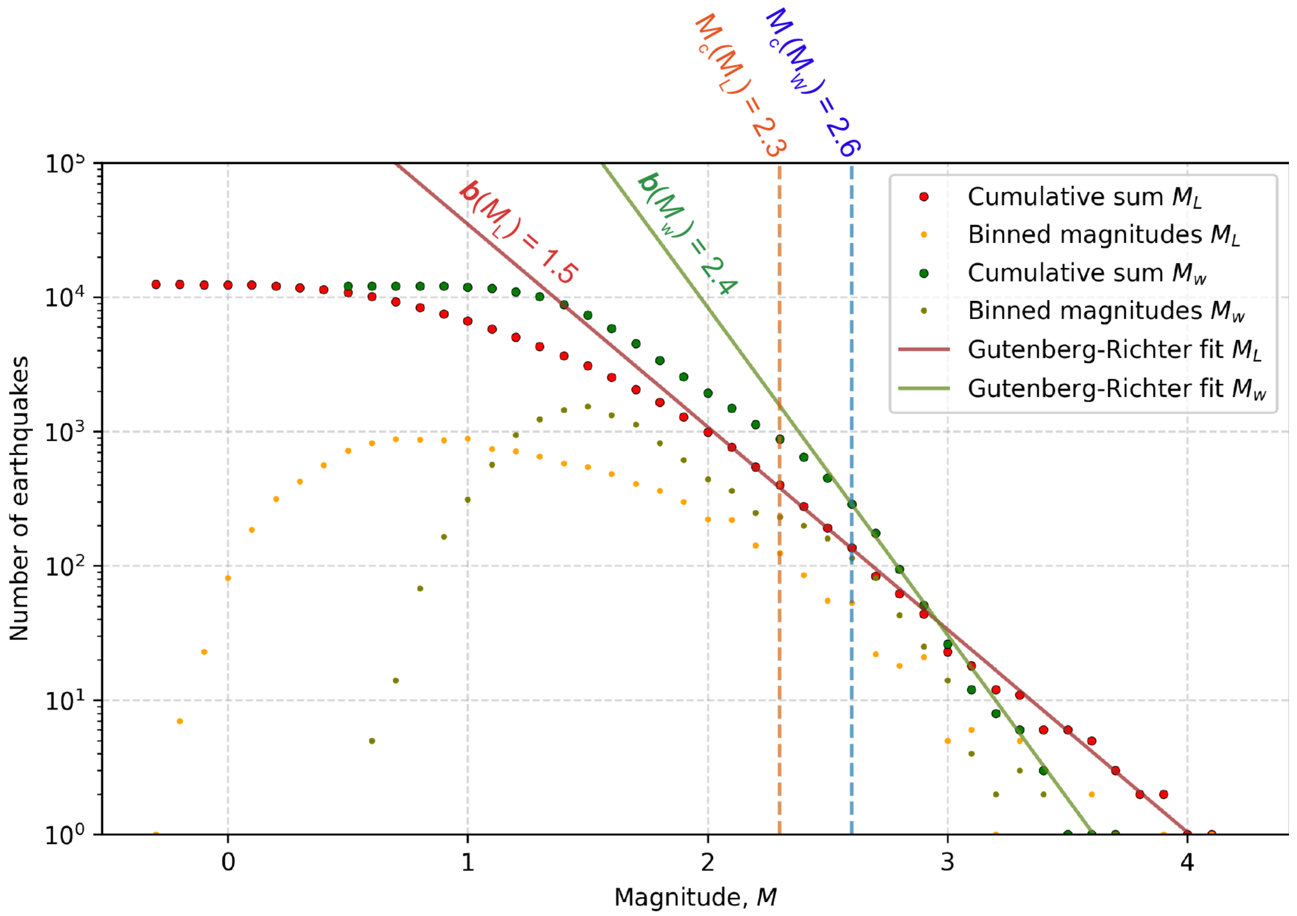

Fig S13: Overall catalogue frequency-magnitude distribution showing computed *b*-value and magnitude of completeness (M_c) from Moment Magnitude (M_w) and Local Magnitudes (M_L), calculated using the boundary-value-stability method[3]. Magnitudes are binned in 0.1 units.

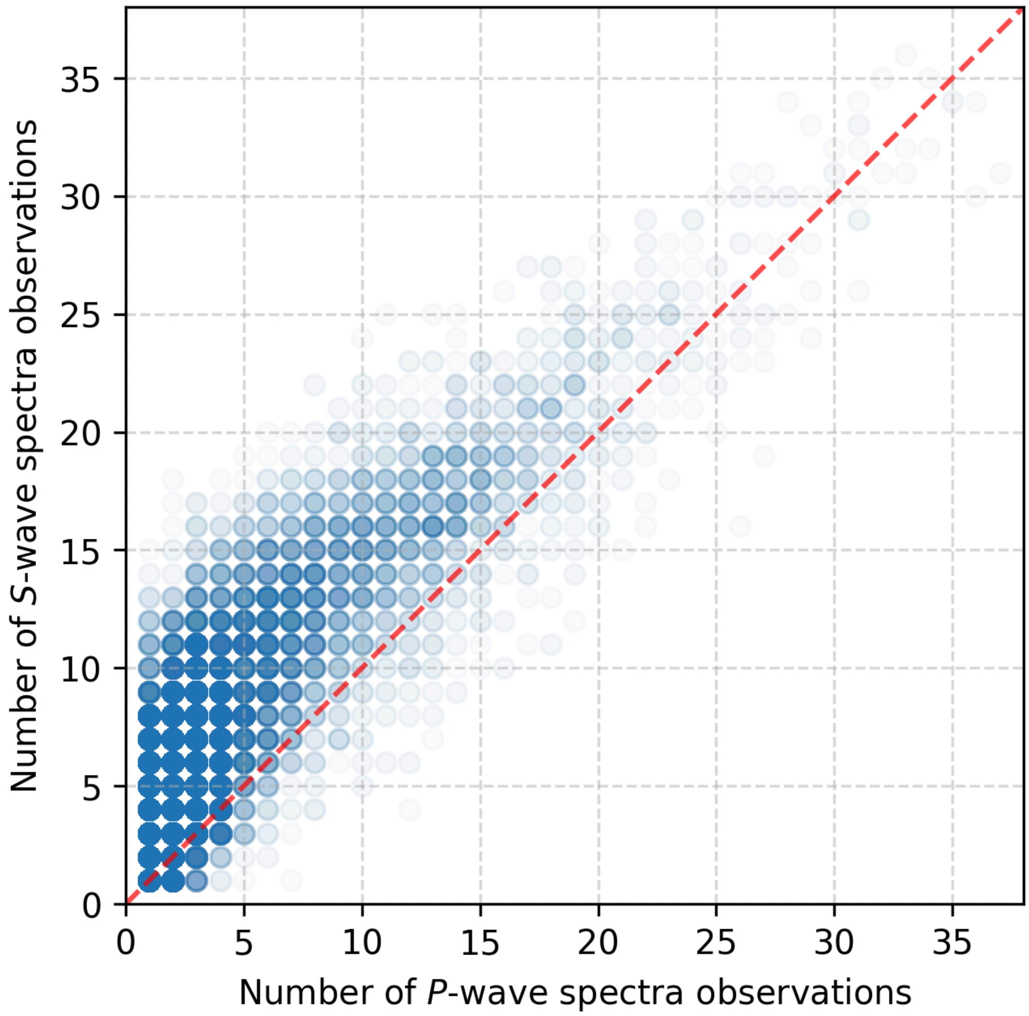

Fig. S14: Comparison between robust station observations per event for SH-wave spectra versus P-wave spectra. Each event is assigned a transparent marker, so the opacity of points reflects the density of observations.

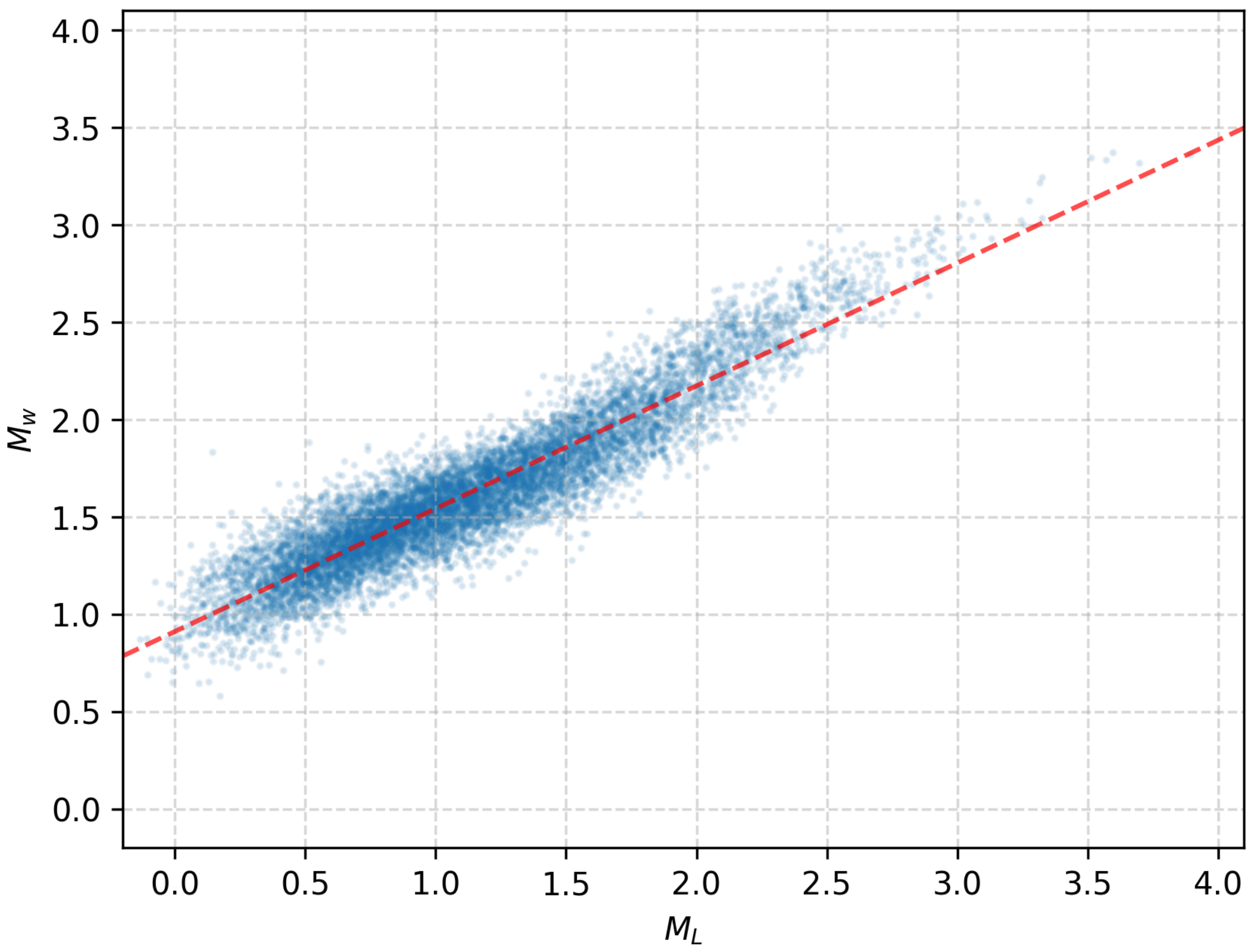

Fig. S15: Comparison between local magnitude (M_L) and moment magnitude (M_w). The dashed red line shows a linear regression fit: $M_w = 0.63M_L + 0.91$.

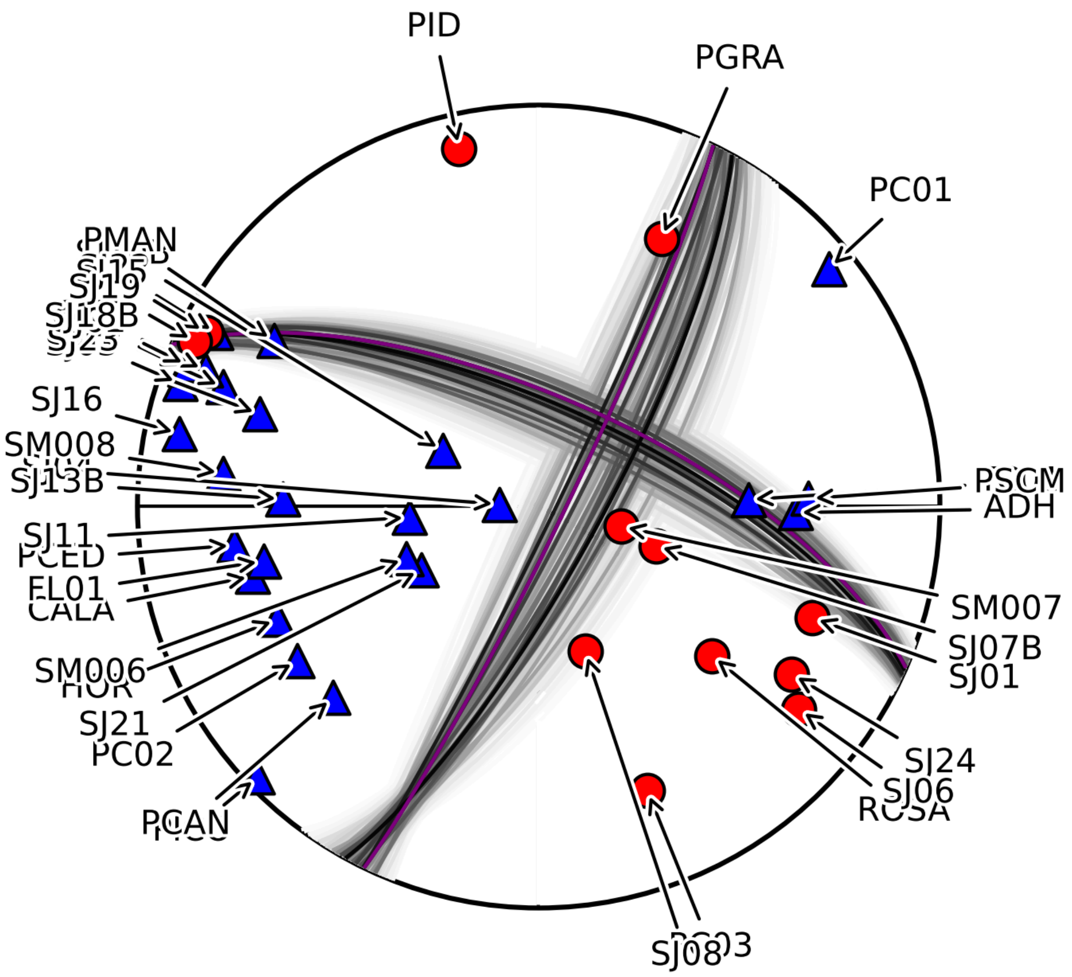

Fig S16: Example of a tightly constrained earthquake focal mechanism based on P-wave polarities. The event had an origin time of 2022-07-28 20:51:23, a depth of 10.2 km, and a moment magnitude of 2.88. Red circles represent compressional first motions, while blue triangles represent dilatational first motions. The range of possible solutions is indicated by the black lines, with the best-fitting solution shown in purple.

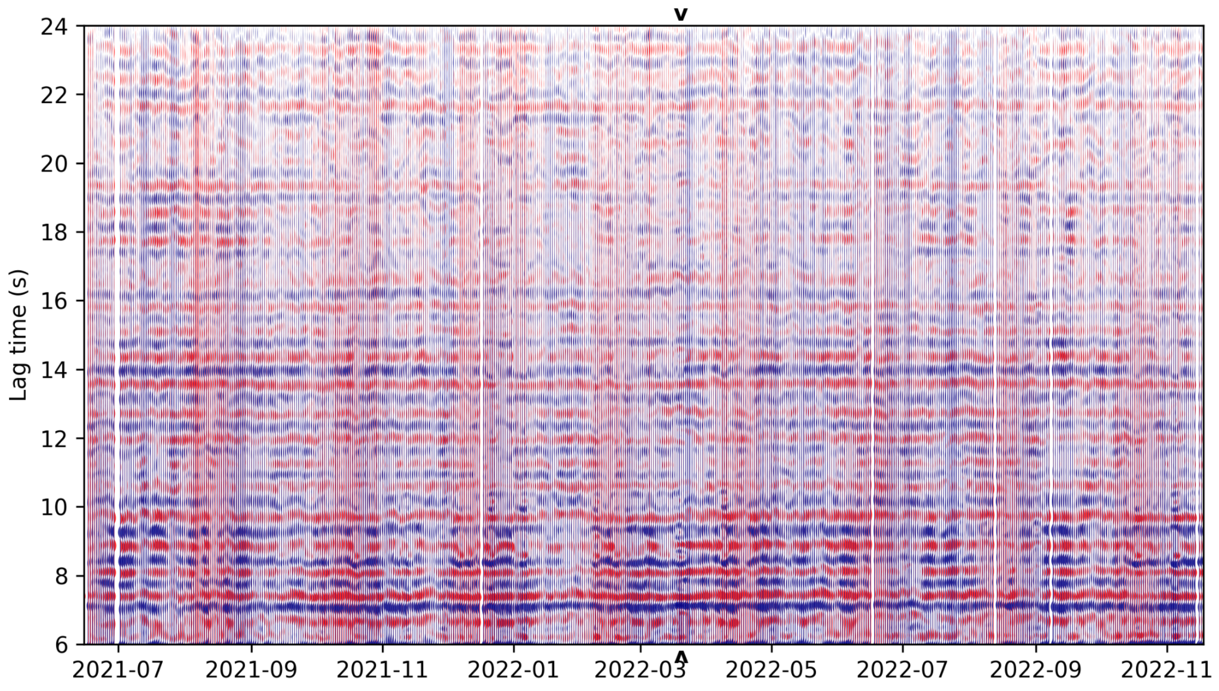

Fig. S17: Same as Fig. 3d, but instead showing the results from station PM.ROSA. The arrowheads above and below the x-axis denote the onset of the seismo-volcanic unrest.

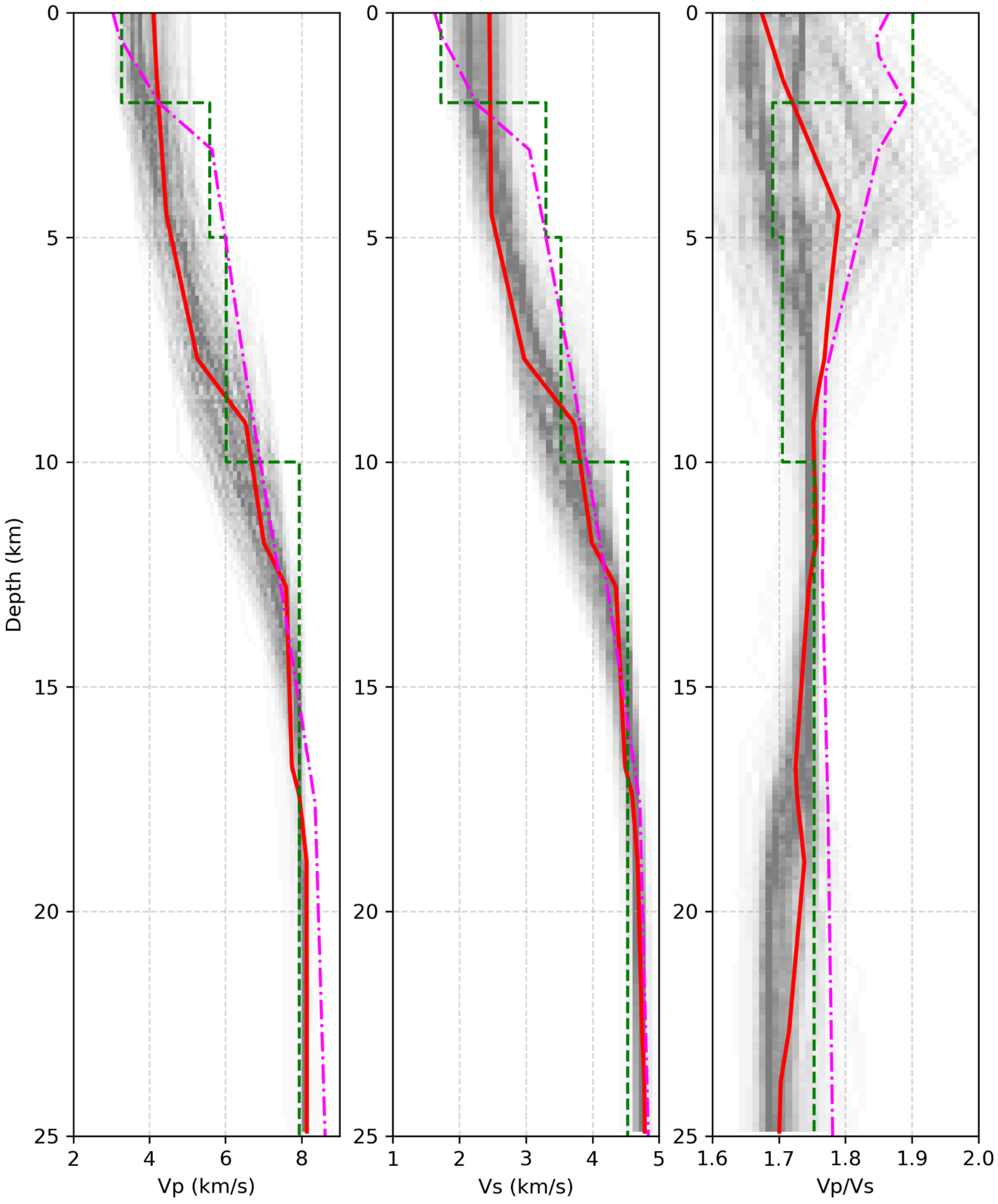

Fig. S18: Minimum 1-D velocity model. Left: P-wave velocity; middle: S-wave velocity; right: v_p/v_s ratio. The red line shows the model with the lowest RMS misfit to arrival time observations. The grey shading shows the range of inverted models that have an RMS misfit within 5% of the best-fitting model. The green dashed line represents the layered model previously obtained by Ferreira et al.[4] using Rayleigh-wave ellipticity measurements at station PM.ROSA, located on the west of São Jorge (Fig. 1b). The magenta dash-dotted line is a 1-D model from using local earthquake arrival times for the Faial-Pico region of the central Azores[5].

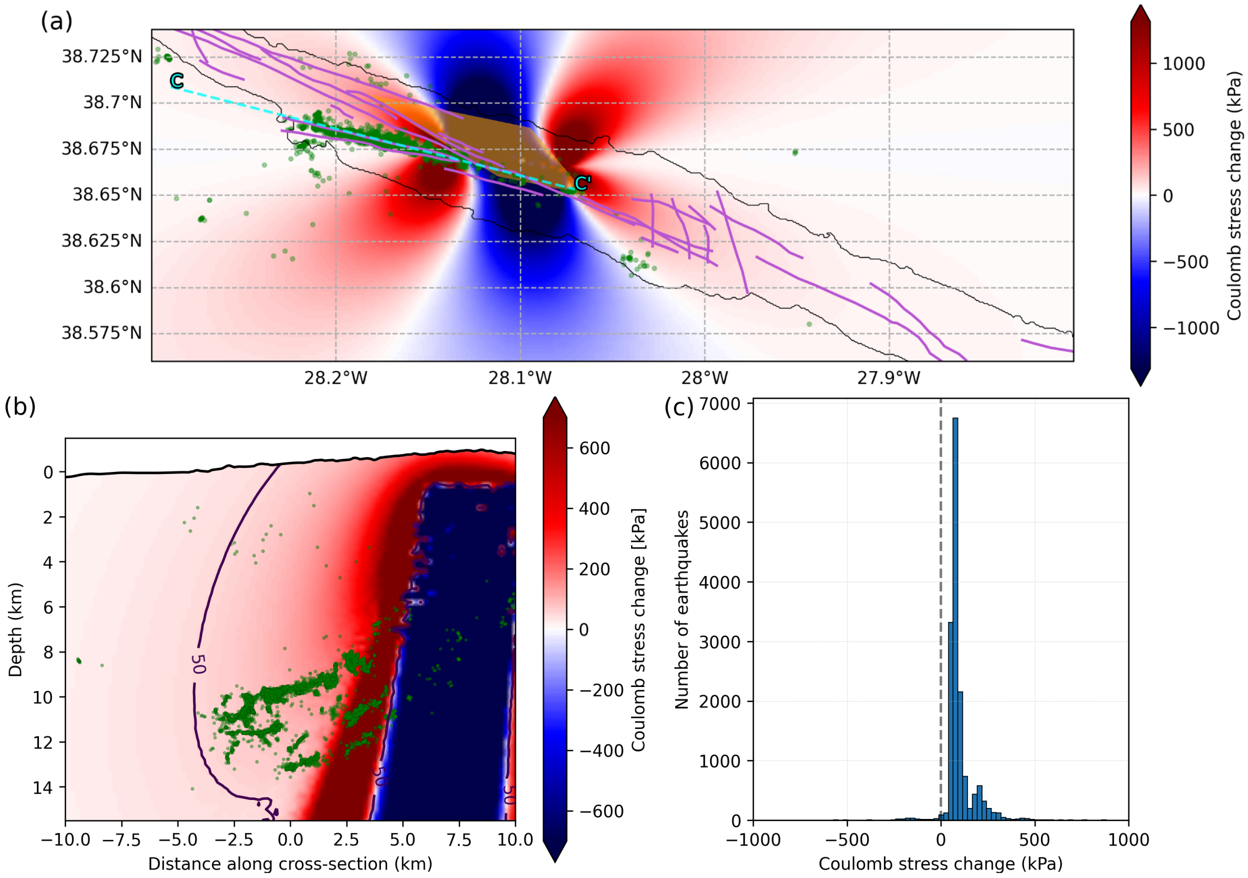

Fig. S19. Coulomb stress analysis due to the modelled dike intrusion. a) Map showing the Coulomb stress change at 10.5 km depth (the mean depth of seismicity), based on vertical-dipping left-lateral (0° rake) receiver faults striking 280° (as indicated by the seismicity locations and focal mechanisms). Pink lines are mapped fault traces; green circles denote relocated earthquake epicentres; orange shading is the modelled dike intrusion. b) Same as (a), with the model plotted along cross-section C-C' striking parallel to the main seismicity lineation. c) Histogram showing the computed Coulomb stress at each earthquake hypocentre. The median value is 87 kPa.

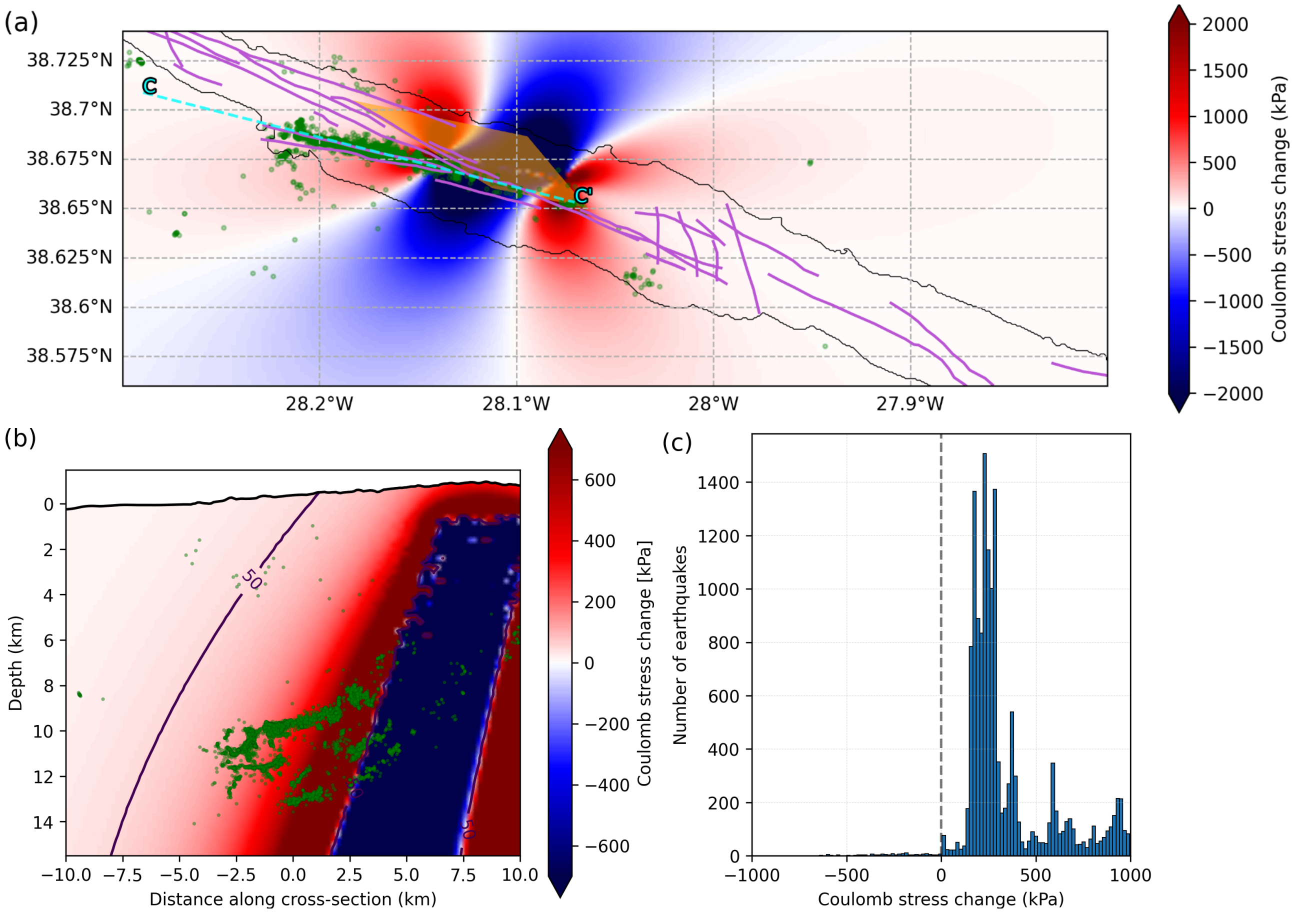

Fig. S20. Same as Fig. S19, except for right-lateral strike-slip failure on receiver faults.

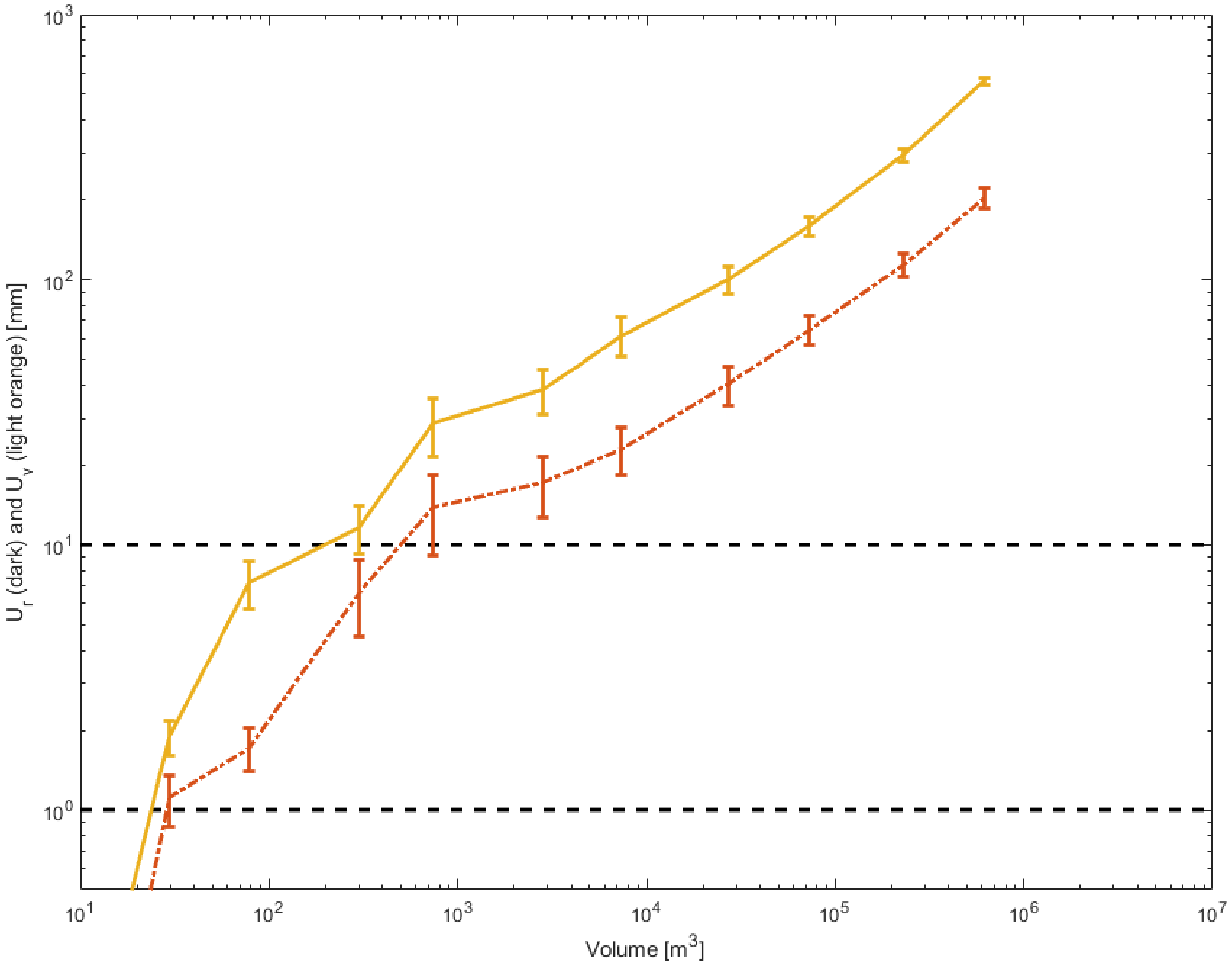

Fig. S21: Geodetic forward modelling tests. To evaluate whether the seismicity could be related to an additional magmatic intrusion, we simulated displacement fields due to 20,000 intrusions injecting in an elastic half-space with variable locations, orientations and sizes-volumes within the depth range of the relocated seismicity (9 to 15 km) to estimate peak horizontal (dark orange) and peak vertical (light orange) displacements due to magmatic injections. Horizontal dashed lines mark displacement values of 1 mm (usual noise level threshold for GNSS detectability) and 1 cm (usual noise level threshold for InSAR detectability). Based on this analysis, we note that only very minor magmatic injections with a volume of less than 10^2-10^3 m^3 would be undetected.

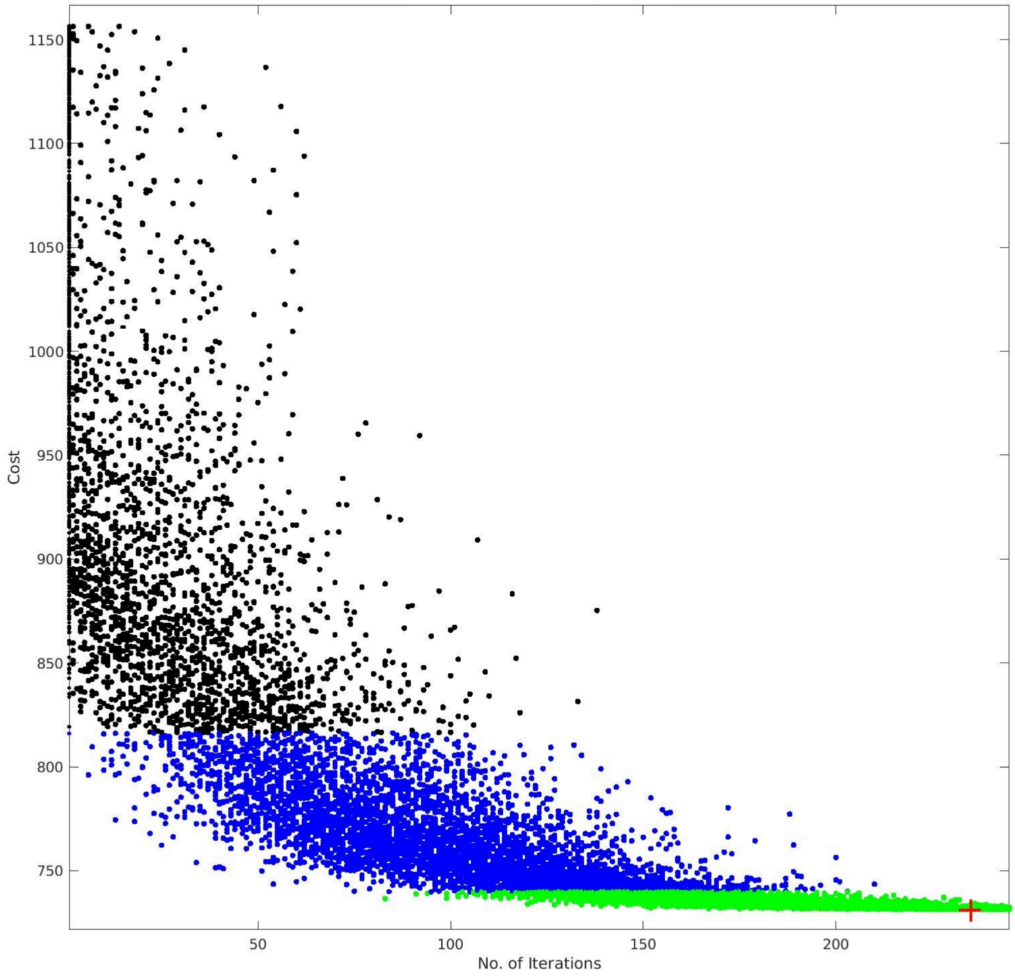

Fig. S22. Convergence of the non-linear inversion for the overall cost function of geometric parameters. The inversion terminated after 250 iterations, the model “cost” fell below 750 and was stable for approximately the last 100 iterations, indicating that the solution had stabilised. Blue points are those <20% of the initial cost function value; green points are those <2% of the initial cost value. The red “+” indicates the final accepted model.

Supplementary Tables

Table S1. Geodetically constrained quadrangle diking model parameter results using GNSS displacement vectors and ascending and descending InSAR observations (Figs S9 & S10). Summary results of the inversion, reporting inversion search bounds, best-fitting model (blue symbols in Figure S11 and S12), mean (red symbols in Figure S11 and S12) and standard deviations of the model parameters based on the probability density functions, and 95% confidence intervals. Coordinates are in metres in UTM zone 26S.

	Geometric parameters								
	Easting (m)	*Northing (m)*	*Top depth (m)*	*Top length (m)*	*Strike (°)*	*Dip (°)*	*Shear angle (°)*	*Length (m)*	*Bottom Length (m)*
**Lower search bounds**	399,000	4,275,000	-5,000	1,000	80	50	-40	1,000	1,000
**Upper search bounds**	413,000	4,282,000	500	40,000	150	130	40	45,000	40,000
**Best-fit model param.**	405,291	4,279,118	-597	4,253	105	97	-11	26,683	8,094
**Mean model param.**	405,829	4,279,237	-844	4,523	105	96	-18	22,843	7,601
**Standard deviation**	1,182	551	580	2,558	7	7	11	7,407	4,698
**Lower confidence interval (5%)**	403,451	4,278,087	-2,032	1,200	92	81	-37	11,666	1,393
**Upper confidence interval (95%)**	408,459	4,280,352	292	10,639	120	108	5	41,888	17,939
**Parameter definition**	East position of the midpoint of the top of the quadrangle	North position of the mid-point of the top of the quadrangle	Depth to the top of the quadrangle	Horizontal length of the top of the quadrangle	Azimuth of the top and bottom axes of the quadrangle using a right-handed coordinate system	Greatest inclination angle Perpendicular (clockwise) to strike, downward along the line of greatest inclination.	Acute angle between the dike's dip direction and the surface line of steepest descent (the line that connects the midpoints of the top and bottom of the quadrangle)	Depth to the bottom of the quadrangle	Horizontal length of the bottom of the quadrangle

Model	RMS residual (mm)		
	GNSS	***InSAR Ascending***	***InSAR Descending***
Single, quadrangular dike [*QD*] (preferred; Table S1)	3.0	9.0	10.6
Rectangular dike [*D*]	5.5	9.5	10.3
Double-Dike Model [*D+D*]	6.6	7.9	12.7
Fault + Dike [*F+D*]	6.6	10.8	15.7
Sheared dike [*SDIKE*]	7.2	12.9	13.5
Mogi + dike [*M+D*]	6.7	7.4	14.0
Hinged Double-Dike [*H*]	3.5	9.5	11.8
Variable-widths Hinged Double-Dike [*W*]	3.5	8.9	10.1
Yang + Dike [*Y+D*]	6.5	12.4	12.3
Single, Dike with only GNSS data [*D-GNSS*]	1.9	-	-
Singular quadrangular dike with vertical curvature [*QDC*]	3.0	8.8	10.5

Table S2: RMS residuals for the different models explored. Values are coloured green and red if they show a >10% improvement or decline, respectively, relative to the preferred model [QD] shown in the first row.

Supplementary Text

Text S1: Exploration of alternative geodetic models

S1.1. Elastic half-space geodetic models with GBIS

To evaluate the source of the 2022 São Jorge deformation, we conducted a comprehensive suite of geodetic inversions using the Geodetic Bayesian Inversion Software (GBIS v1.1 [6]). Our objective was to test whether alternative geometries, other than a single planar dike, could plausibly reproduce the observed GNSS and InSAR displacements.

We implemented a range of analytical source models, including: Mogi [***M***]: Point pressure source; Yang [***Y***]: prolate spheroid; Okada Dike: rectangular tensile dislocation; Okada Fault [***F***]: rectangular fault with uniform slip; Sheared Dike [***SDIKE***]: dike with tensile and strike-slip components; composite models [e.g., ***D+F***, ***D+D***, ***D+T***, ***Y+D***, ***M+D***]; custom geometries [***H, W***, ***QDC***]: hinged, segmented and curved dike configurations.

All models, except a single GNSS-only inversion [***QD-GNSS***], were tested on the same GNSS and Sentinel-1 ascending- and descending-InSAR datasets, using consistent preprocessing and variogram-based InSAR data error modelling. Inversions were performed using a Metropolis-Hastings Markov Chain Monte Carlo (MCMC) approach with 1e6 iterations, and convergence was assessed using posterior density and trace plots.

A single dike model is sufficient to explain the observations. A near-vertical dike (~89° dip), striking parallel to the PdC-FZ fault zone (~282°), with an opening of approximately 5.6 m and a top depth of around 3 km, provides the best fit to both GNSS and InSAR data. This model yields minimal residuals and physically most plausible geometry, outperforming all other tested configurations. Inversion of just GNSS data is consistent with a single dyke. Still, its geometry (strike and lateral position) is poorly constrained, underscoring the importance of InSAR data for resolving near-surface deformation.

Although some alternative composite models fit the observations comparably, they either fail to reproduce the symmetry and amplitude of the InSAR lobes or the far-field extension recorded by the GNSS network, introduce unrealistic trade-offs, or are geologically implausible. Such models include: Double Dike (*D+D*); Fault + Dike (*F+D*); Sheared Dike (*SDIKE*); Mogi + Dike (*M+D*); Yang + Dike (*Y+D*). We also find that hinged and segmented dike models are typically dominated by a single source (H or W), rendering the second segment unnecessary. These models introduced additional model degrees of freedom (e.g., curvature, variable dip), but did not significantly improve the fit or resolve new features.

While complex plumbing systems are typical in volcanic settings, geodetic data, given their limited coverage, do not support the existence of other elastically deforming deep (i.e., mantle) or shallow crustal sources/magma reservoirs before or after the most intense phase of deformation and seismicity.

1.1 Rectangular Dike Model [*D*]

Model geometry. Dike with variable length, width, depth, dip, strike, and uniform opening (Okada-type dike).

Table S1.1: Inversion results for the rectangular dike model [*D*]								
**Parameter**	***Length (m)***	***Width (m)***	***Depth (m)***	***Dip (°)***	***Strike (°)***	***X (m)***	***Y (m)***	***Opening (m)***
**Optimal value**	2,522	523	3,040	–88.7	282.1	–4,810	1,817	5.60
**95% confidence interval**	[647 – 4,185]	[263 – 2,376]	[2,587 – 3,322]	[–89.8 – –84.5]	[276.8 – 285.5]	[–5,004 – –4,357]	[1,628 – 2,038]	[1.81 – 9.71]

Interpretation. This model yields a near-vertical dike (dip ~89°) with a moderate depth (~3 km) and a relatively narrow width (~500 m), consistent with a shallow crustal intrusion. The strike (~282°) aligns well with the regional tectonic fabric and the mapped PdC-FZ fault zone. The opening magnitude (~5.6 m) is physically plausible given the observed surface displacements. Compared to more complex models (e.g., *SDIKE* or composite sources), this simple dike model provides a better fit to the InSAR data (lower RMS) and avoids introducing unnecessary complexity or parameter trade-offs. The posterior distributions are well-constrained, and the inversion converges well. These results support the interpretation that a single, vertically extensive dike is the dominant source of deformation during the 2022 São Jorge unrest.

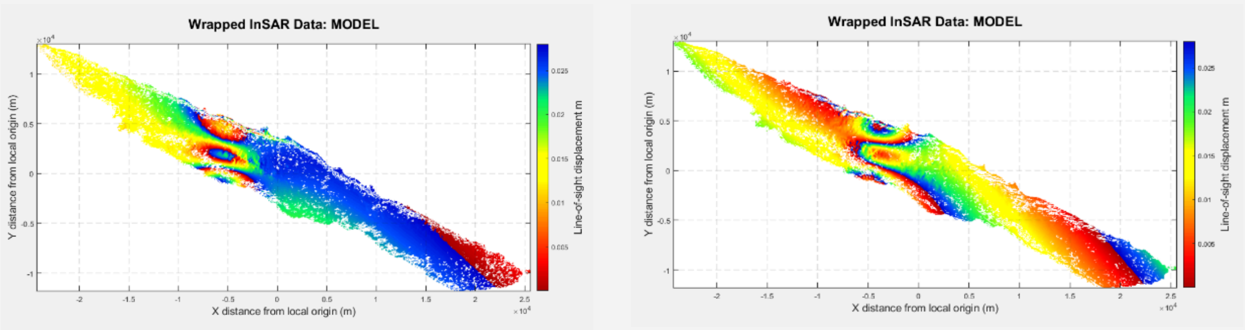

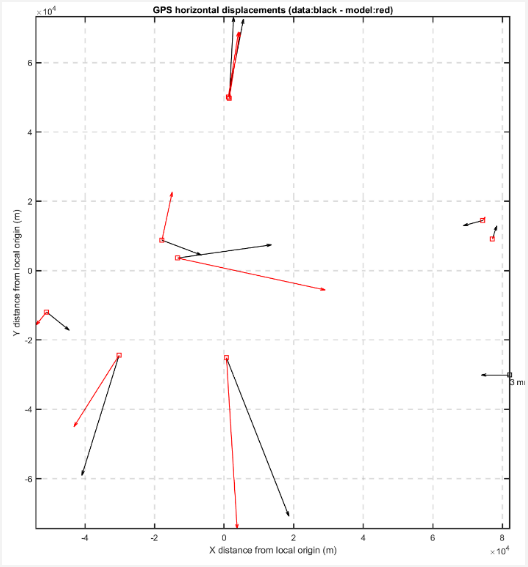

Figure S23. Single dike model [D] simulated displacements. Top-left: ascending interferogram; top-right: descending interferogram; bottom-left: modelled (red) and observed (black) GNSS displacements.

1.2 Double-Dike Model [*D*+*D*]

Model geometry: 2 dikes with variable length, width, depth, dip, strike, and opening.

Table S1.2: **Inversion results for the double-dike model [*D*+*D*]**					
**Dike 1 (Shallower, ESE-striking)**			**Dike 2 (Deeper, WNW-striking)**		
**Parameter**	**Optimal Value**	**95% confidence interval**	**Parameter**	**Optimal Value**	**95% confidence interval**
Length (m)	5,727	[4,515 – 6,493]	Length (m)	5,428	[4,987 – 5,618]
Width (m)	326	[106 – 1,032]	Width (m)	119	[102 – 472]
Depth (m)	1,212	[1,010 – 1,720]	Depth (m)	638	[520 – 826]
Dip (°)	–4.0	[–60.7 – –0.6]	Dip (°)	–0.11	[–3.26 – –0.12]
Strike (°)	121.1	[112.4 – 126.7]	Strike (°)	296.3	[294.0 – 297.9]
Opening (m)	0.33	[0.11 – 1.59]	Opening (m)	0.42	[0.11 – 0.55]

Interpretation. The double-dike model yields a slightly improved fit to the ascending-track InSAR data compared with the single-dike model. However, the inferred geometry is problematic. (1) Both dikes are very shallow (depths < 2 km), inconsistent with the broader deformation pattern and the lack of shallow seismicity. (2) The dips are nearly horizontal, which is mechanically implausible for dike intrusions and inconsistent with the observed vertical deformation gradient. (3) The strikes of the two dikes differ significantly (121° vs. 296°), suggesting a physically disconnected or unrealistic configuration. While the model achieves a marginally better statistical fit, the resulting geometry lacks geological plausibility. Therefore, we interpret this result as an overfit to localised surface signals rather than a physically meaningful improvement over the simpler vertical dike model.

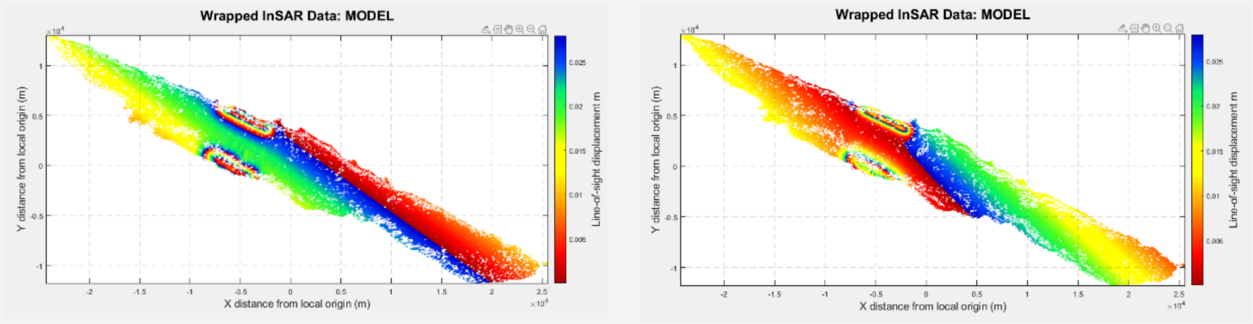

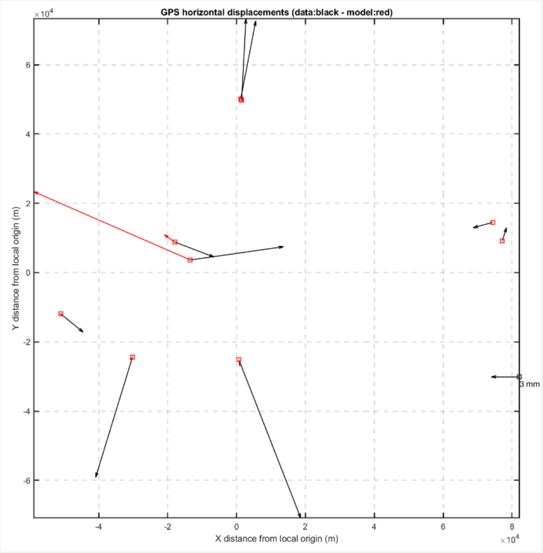

Figure S24. Double dike model [D+D] simulated displacements. Top-left: ascending interferogram; top-right: descending interferogram; bottom-left: modelled (red) and observed (black) GNSS displacements,

1.3 Fault + Dike composite model [*F*+*D*]

Model geometry: Fault as rectangular dislocation with strike-slip and dip-slip components; and a dyke as a rectangular tensile dislocation with variable geometry and uniform opening.

Interpretation: The F+D model introduces a fault component with a shallow dip and modest left-lateral strike-slip motion. The dike component is long and shallow, with a nearly horizontal dip and a moderate opening. While this model allows for a dual-source interpretation, it does not improve the fit compared to the single-dike model, and it introduces several issues: 1) The fault geometry is poorly constrained (wide range of depths and dips), and the inferred slip is small and uncertain. 2) The dike geometry is shallow and nearly horizontal (mechanically implausible). 3) The InSAR misfits are high. Overall, the F+D model is not supported by the data.

Table S1.3: **Inversion results for the fault+dike composite model [*F*+*D*]**

Fault			Dike		
**Parameter**	**Optimal Value**	**95% confidence interval**	**Parameter**	**Optimal Value**	**95% confidence interval**
Length (m)	9,685	[2,500 – 12,485]	Length (m)	22,044	[11,120 – 22,114]
Width (m)	11,139	[3,004 – 12,374]	Width (m)	111	[102 – 751]
Depth (m)	869	[631 – 10,778]	Depth (m)	1,014	[894 – 1,580]
Dip (°)	–13.9	[–45.5 – –10.3]	Dip (°)	–0.15	[–8.56 – –0.14]
Strike (°)	359.7	[355.7 – 360.0]	Strike (°)	295.3	[292.4 – 296.4]
Strike-slip (m)	–0.22	[–3.85 – –0.18]	Opening (m)	0.90	[0.22 – 1.30]
Dip-slip (m)	~0.01	[–0.008 – 0.010]			

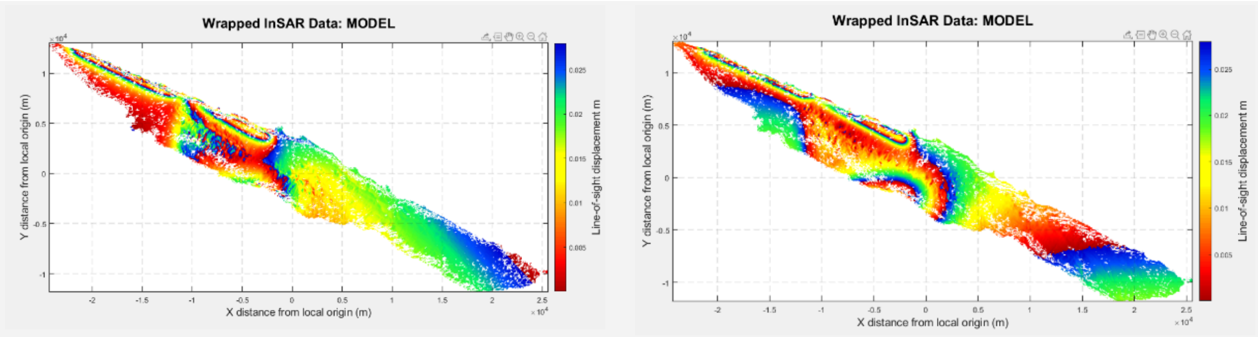

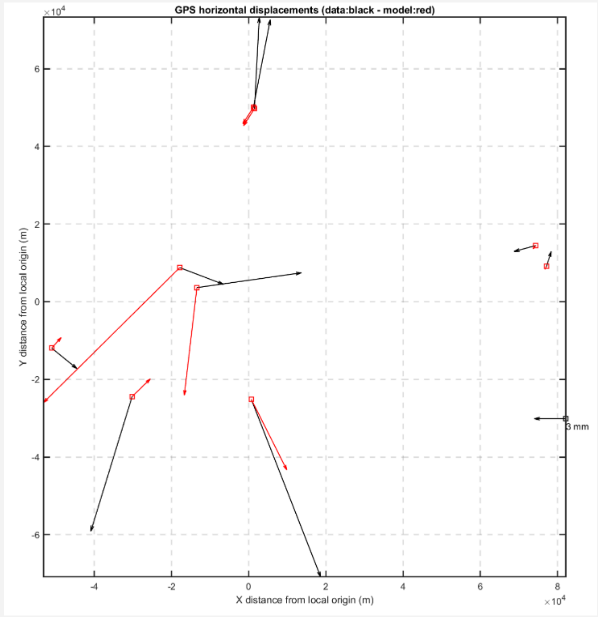

Figure S25. Dike + fault composite model [D+F] simulated displacements. Top-left: ascending interferogram; top-right: descending interferogram; bottom-left: modelled (red) and observed (black) GNSS displacements,

1.4 Sheared dike model [*SDIKE*]

Model geometry. Rectangular dislocation with variable length, width, depth, dip, strike, and both strike-slip and opening components.

Table S1.3: **Inversion results for the sheared dike model [*SDIKE*]**										
**Parameter**	***Length (m)***	***Width (m)***	***Depth (m)***	***Dip (°)***	***Strike (°)***	***X (m)***	***Y (m)***	***Strike-sli p (m)***	***Dip-slip (m)***	***Opening (m)***
**Optimal value**	19,590	118	955	-0.36	294.4	-9,585	6,802	1.05	~0	0.49
**95% confidence interval**	[18,321 – 29,902]	[101 – 618]	[681 – 1,679]	[–28.6 – –0.13]	[293.5 – 295.6]	[–14,270 – –8,849]	[6,399 – 8,966]	[0.15 – 1.99]	[–0.009 – 0.009]	[0.08 – 1.01]

Interpretation: While the SDIKE model provides a reasonable fit to the data, the inferred geometry is very shallow (top edge ~950 m depth), with a nearly horizontal dip (–0.36°), and a narrow width (~118 m). These characteristics are inconsistent with the broader, vertically extensive deformation pattern observed in InSAR and GNSS data. Additionally, the significant strike-slip component (1.05 m) is not supported by the seismicity distribution, which is dominantly strike-slip but not co-located with the modelled dislocation. Therefore, despite its flexibility, the SDIKE model does not significantly improve fit relative to simpler dike models and introduces geometric inconsistencies. This result supports our interpretation that a vertically extensive, near-vertical dike remains the most plausible source.

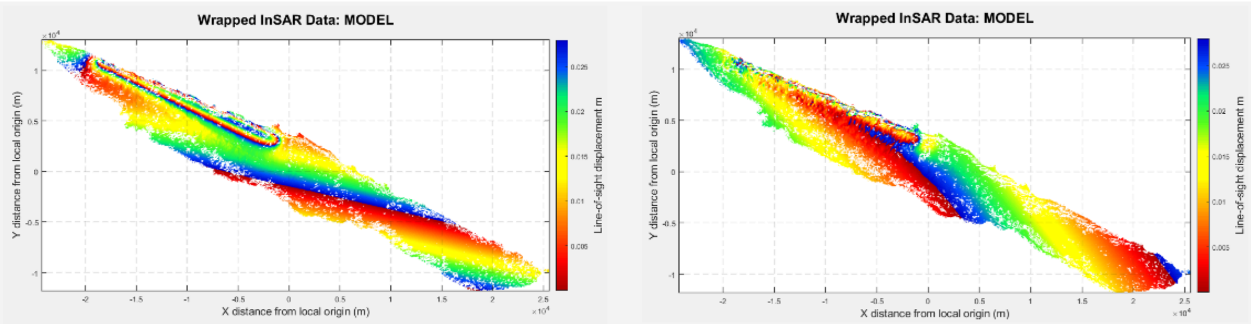

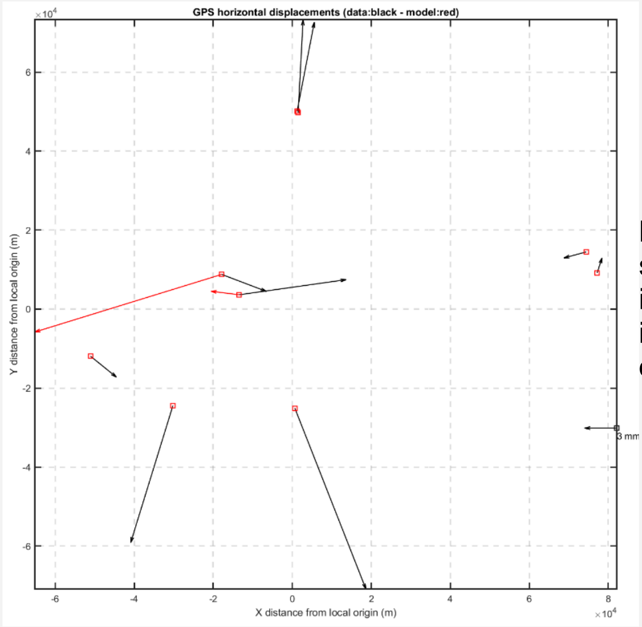

Figure S26. Shear dike model [SDIKE] simulated displacements. Top-left: ascending interferogram; top-right: descending interferogram; bottom-left: modelled (red) and observed (black) GNSS displacements,

1.5 Mogi + Dike Composite Model [*M+D*]

Model geometry: Mogi source as a deep point pressure source with variable depth and volume change, and a shallow dyke as a rectangular tensile dislocation with variable geometry and opening.

Table S1.4: **Inversion results for the Mogi + dike composite model [*M+D*]**

**Mogi Source**			**Dike Component**		
**Parameter**	**Optimal Value**	**95% confidence interval**	**Parameter**	**Optimal Value**	**95% confidence interval**
X (m)	–4,799	[–5,111 —4,680]	*Length (m)*	5,097	[4,782 – 10,438]
Y (m)	–1,230	[–1,789 – –829]	*Width (m)*	847	[411 – 1,344]
Depth (m)	1,636	[1,294 – 2,563]	*Depth (m)*	102	[100 – 347]
Volume change (m³)	610,161	[335,618 1,610,143]	*Dip (°)*	–74.9	[–89.8 – –72.6]
			Strike (°)	116.6	[107.6 – 117.3]
			Opening (m)	0.11	[0.10 – 0.35]

Interpretation: The M+D model achieves a good fit to the data, particularly for the ascending InSAR track. However, the inferred geometry presents several issues: 1) The Mogi source is relatively shallow (~1.6 km), which is inconsistent with typical deep magma reservoirs and with the lack of deep seismicity. 2) The dike is also very shallow (~100 m depth), with a steep dip and modest opening, which may not be resolvable given the spatial resolution of the data. 3) The strike of the dike (~116°) is oblique to the regional tectonic fabric and inconsistent with the mapped fault structures. While the model performs well statistically, the physical interpretation is less compelling. The shallow depths of both sources and the oblique geometry suggest that the model may be overfitting localised surface signals rather than capturing a realistic magmatic process. The simpler single-dike model remains more consistent with the geological and geophysical context.

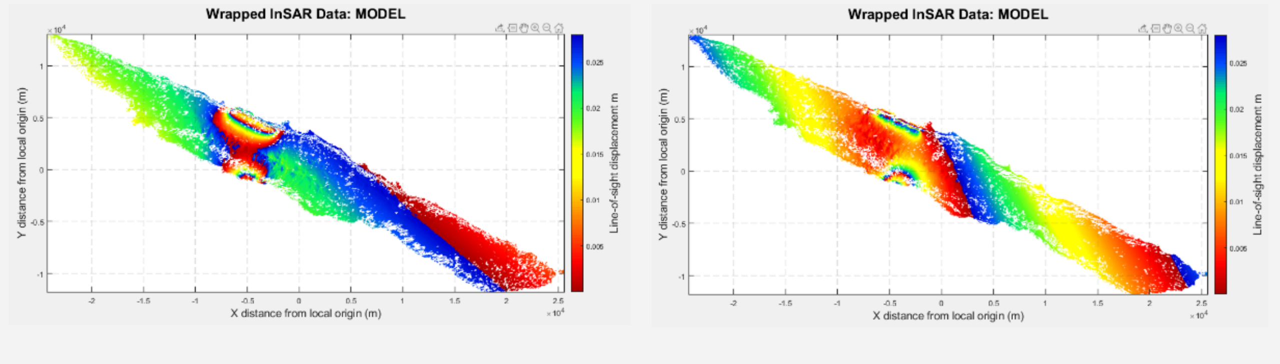

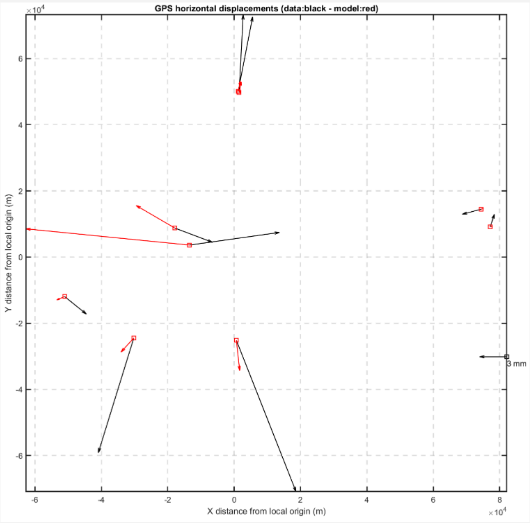

Figure S27. Mogi + dike model [M+D] simulated displacements. Top-left: ascending interferogram; top-right: descending interferogram; bottom-left: modelled (red) and observed (black) GNSS displacements.

1.6 Hinged Double-Dike Model [*H*]

Model geometry: Two connected dike segments with independent dips, widths, and openings, but with the same strike and length, simulating a kinked or stepped intrusion path.

Table S1.5: **Inversion results for the hinged double-dike model [*H*]**

**Dike Segment 1 (Shallow, north dipping)**			**Dike Segment 2 (Deeper, steeper)**		
**Parameter**	**Optimal value**	**95% confidence interval**	**Parameter**	**Optimal value**	**95% confidence interval**
Length (m)	2,165	[346 – 3,232]			
Width (m)	244	[188 – 3,665]	*Width (m)*	47,255	[365 – 49,277]
Depth (m)	2,938	[2,249 – 3,208]	*Dip (°)*	-89.8	[–89.8 – –48.0]
Dip (°)	–87.1	[–89.8 – –39.3]	*Opening (m)*	0.51	[0.03 – 9.11]
Opening (m)	8.84	[0.19 – 9.83]	*Strike (°)*	282.7	[277.6 – 285.3]

Interpretation. The hinged double-dike model provides a reasonable fit to the data, with RMS values comparable to the single-dike model. However, the geometry inferred is problematic. The opening of the first segment is larger (~8.8 m) than in our preferred model, suggesting substantial strain for a shallow intrusion. While the model captures some complexity in the data, it lacks physical plausibility in specific parameters. Nevertheless, this model is similar to the simpler single-dike model that includes topography.

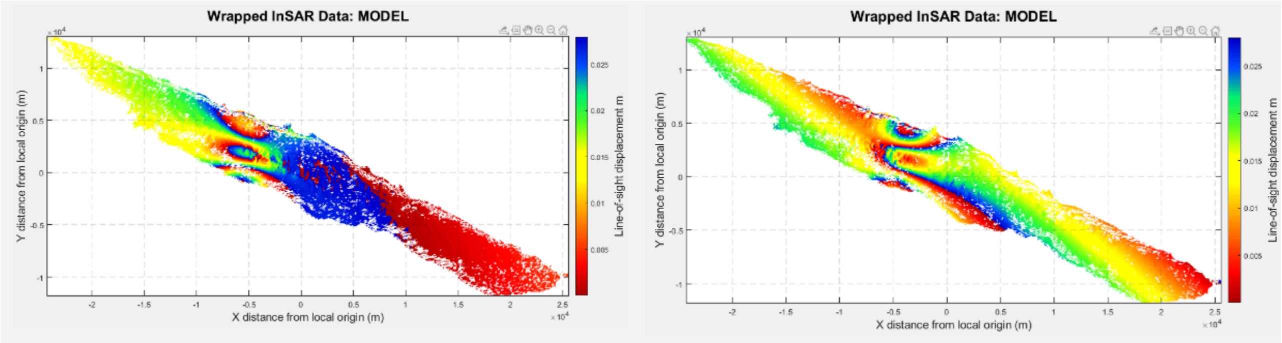

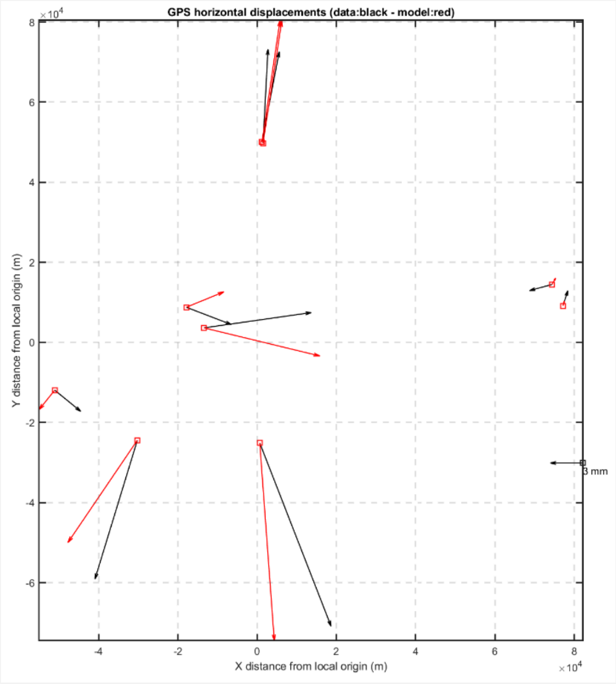

Figure S28. Hinged double-dike model [H] simulated displacements. Top-left: ascending interferogram; top-right: descending interferogram; bottom-left: modelled (red) and observed (black) GNSS displacements.

1.7 Variable-widths Hinged Double-Dike Model [*W*]

Model geometry. Two dike segments with independent geometries and openings, sharing a common strike, simulating a kinked or stepped dike with lateral and vertical complexity.

Table S1.6: Inversion results for the Hinged Double-Dike Model [*W*]					
**Dike Segment 1 (Shallower, shorter)**			**Dike Segment 2 (Deeper, longer)**		
**Parameter**	**Optimal value**	**95% confidence interval**	**Parameter**	**Optimal value**	**95% confidence interval**
Length (m)	1,872	[632 – 3,805]	*Length (m)*	29,651	[1,299 - 48,920]
Width (m)	336	[245 – 2,158]	*Width (m)*	48,947	[1,255 - 49,551]
Depth (m)	3,004	[2,470 – 3,235]	*Dip (°)*	-89.4	[–89.9 – 60.9]
Dip (°)	–88.0	[–89.7 – –83.1]	*Opening (m)*	0.042	[0.017-0.255]
Opening (m)	9.84	[1.56 – 9.83]			

Interpretation. This model achieves a good fit to the data, comparable to the best-performing single-dike models. However, the inferred geometry raises several concerns: 1) The opening of the second segment is tiny (~4 cm), so it contributes minimally to the surface deformation. 3) The first segment dominates the deformation signal, with a large opening (~9.8 m) and a well-constrained geometry. While the model captures some complexity and performs well statistically, the addition of a second segment with a different dip and length is not required by the data.

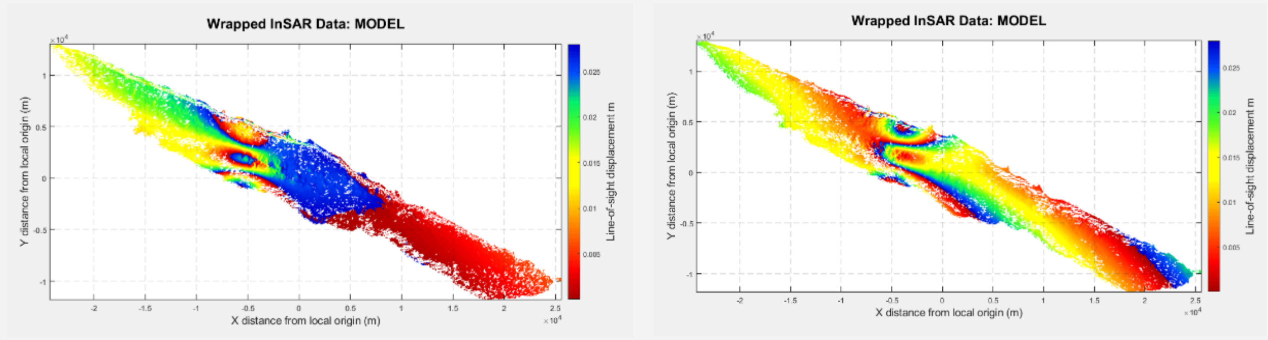

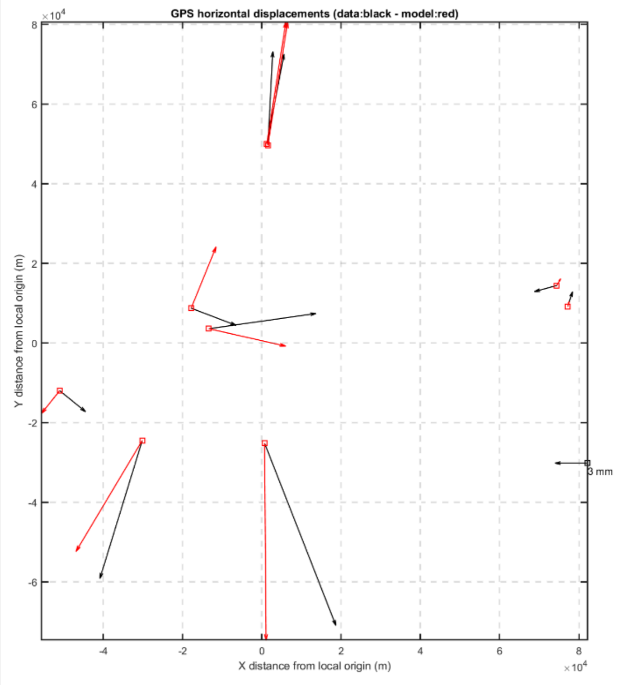

Figure S29. Variable-width hinged double-dike model [W] simulated displacements. Top-left: ascending interferogram; top-right: descending interferogram; bottom-left: modelled (red) and observed (black) GNSS displacements.

1.8 Yang + Dike composite model [*Y*+*D*]

Model geometry. A Yang source as a prolate spheroid representing a pressurised magma body with variable depth, orientation, and pressure; and an Okada-type dyke as a rectangular tensile dislocation with variable geometry and uniform opening.

Table S1.7: **Inversion results for the Yang + Dike composite model [*Y*+*D*]**

Yang Source			Dike Component		
**Parameter**	**Optimal value**	**95% confidence interval**	**Parameter**	**Optimal value**	**95% confidence interval**
X; Y (m)	–3,367; 4,670	[–4,404 – –2,727]; [4,460 – 5,733]	*Length (m)*	24,263	[21,404 – 24,907]
			Width (m)	106	[105 – 835]
Depth (m)	1,657	[1,075 – 2,347]	*Depth (m)*	344	[196 – 384]
Maj. axis (m)	106	[33 – 241]	*Dip (°)*	–60.1	[–70.9 – –33.3]
Aspect Ratio	0.041	[0.011 – 0.143]	*Strike (°)*	288.6	[288.0 – 289.3]
Strike (°)	65.7	[2.4 – 353.3]	*Opening (m)*	2.76	[0.42 – 6.30]
Plunge (°)	–74.4	[–88.9 – –54.8]			
ΔP/μ	114.4	[23.3 – 939.9]			

Interpretation: The Y+D model provides a reasonable fit to the data, with RMS values comparable to other composite models. However, several issues arise: 1) The Yang source is relatively shallow (~1.7 km) and small in volume, with a poorly constrained strike and aspect ratio, suggesting limited contribution to the deformation. 2) The dike dominates the deformation signal, with a considerable length and moderate opening, consistent with other models. While the model is statistically acceptable, the Yang component does not add meaningful explanatory power. The simpler single-dike model remains more geologically plausible and better constrained.

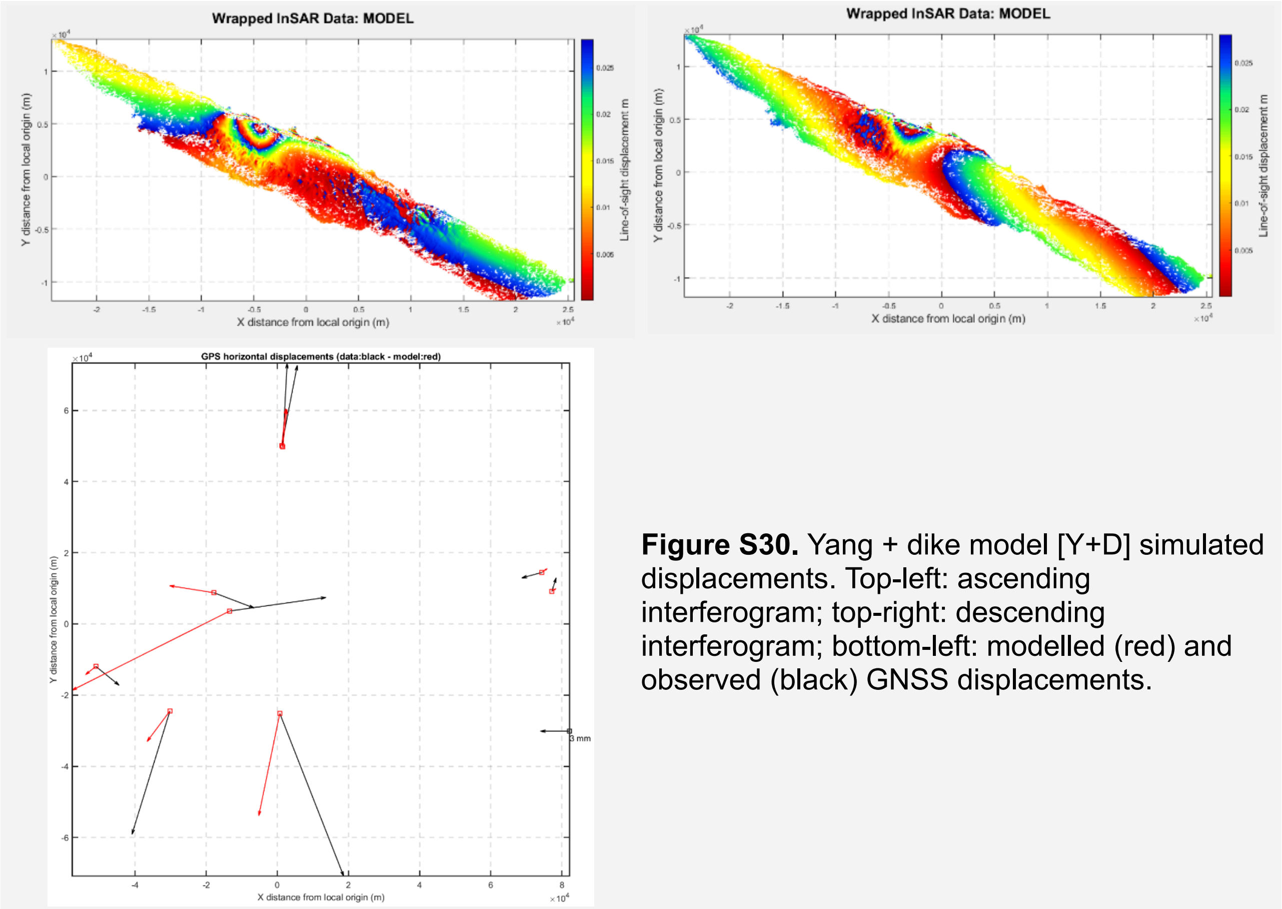

Figure S30. Yang + dike model [Y+D] simulated displacements. Top-left: ascending interferogram; top-right: descending interferogram; bottom-left: modelled (red) and observed (black) GNSS displacements.

1.9 GNSS-Only Dike Model [*D-GNSS*]

Model geometry. Rectangular tensile dislocation (Okada-type) with variable length, width, depth, dip, strike, and uniform opening to test the sensitivity of the GNSS network to the geometry and depth of the magmatic intrusion.

Table S1.8: **Inversion results for the GNSS-Only Dike Model [*D-GNSS*]**

**Parameter**	***Length (m)***	***Width (m)***	***Depth (m)***	***Dip (°)***	***Strike (°)***	***X (m)***	***Y (m)***	***Opening (m)***
**Optimal value**	5,592	1,688	7,846	–89.5	269.1	–9,317	5,777	5.85
**95% confidence interval**	[592 – 23,156]	[663 – 19,058]	[1,560 – 19,387]	[–89.5 – –35.7]	[67.9 – 299.3]	[–13,642 – 9,634]	[–7,084 – 12,532]	[0.31 – 9.33]

Interpretation: The GNSS-only inversion yields a good fit to the GNSS observations with: 1) A deep dike (~7.8 km), but with a wide uncertainty range extending into the upper mantle. 2) An extensive range of strikes and locations, indicating poor constraint on lateral geometry when using only GNSS observations. These results highlight the simplicity of the required model to fit the observations. However, the sparse station distribution and limited sensitivity to localised deformation make it difficult to resolve the intrusion geometry near the surface from GNSS data. The addition of InSAR data significantly enhances the model's resolution and robustness, as demonstrated by the joint inversions.

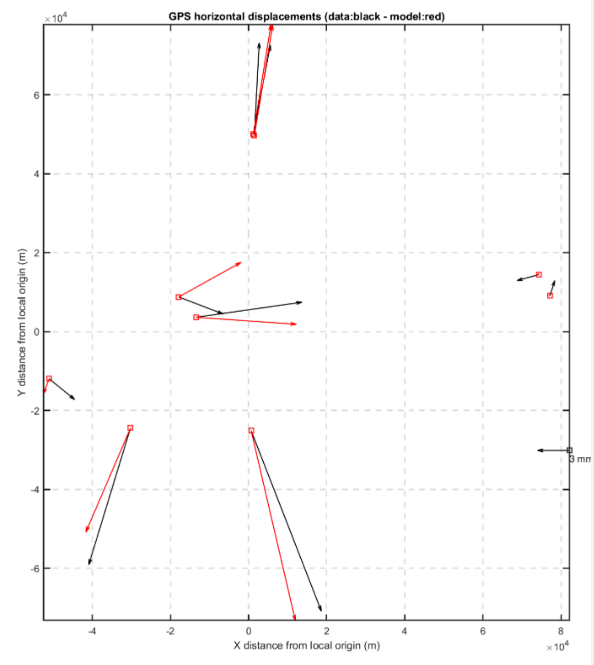

Figure S31. GNSS-Only dike model [D-GNSS] simulated displacements. Top-left: ascending interferogram; top-right: descending interferogram; bottom-left: modelled (red) and observed (black) GNSS displacements.

1.10: Extended BEM inversion allowing for dyke vertical curvature [*QDC*]

To assess whether a more realistic, non-planar geometry could improve the fit to the observed deformation, we invert for the geometry using the Boundary Element Method (BEM), allowing for vertical curvature in the modelled dike. This approach accounts for potential bending or segmentation of the intrusion, which may arise due to heterogeneous stress fields or structural controls.

We again employed the 3D mixed BEM inversion, incorporating realistic topography and bathymetry, using a quadrangular-crack representation with variable curvature. The inversion employed a neighbourhood algorithm to explore a vast parameter space that included strike, dip, and shear angle, top and bottom depths, length, and vertical curvature.

All three components of GNSS displacement vectors and both ascending- and descending-Sentinel-1 InSAR datasets were used, with normalised weights to avoid bias toward any single dataset.

Fig. S2.10 shows the 1D marginal posterior probability density functions (PPDs) for the curvature-enabled BEM inversion. Key observations include that the strike and dip are well constrained, with narrow distributions centred around ~282° and ~83–85°, respectively, consistent with the mapped PdC-FZ fault orientation. The shear angle exhibits a unimodal distribution near zero, indicating a pronounced tendency for the dyke to deepen toward the west. Length and bottom-level parameters exhibit broader distributions, reflecting uncertainty in the deeper geometry because surface displacements are insensitive to deeper features. The vertical curvature and topographic inclination are only moderately constrained, suggesting that a slightly curved geometry may better accommodate the observed displacements.

The comparison between the maximum PPD model (blue) and the mean model (red) indicates good agreement across most parameters, supporting the inversion's convergence and robustness.

Interpretation. Allowing for curvature in the BEM inversion yields a model that is consistent with the simpler planar dike geometry and yields subtle improvements in the fit to GNSS and InSAR data. The inferred curvature is modest and does not fundamentally alter the interpretation of a vertically extensive, near-vertical dike. Nonetheless, the improvement in fit is marginal, and the simpler planar model remains a valid and parsimonious representation.

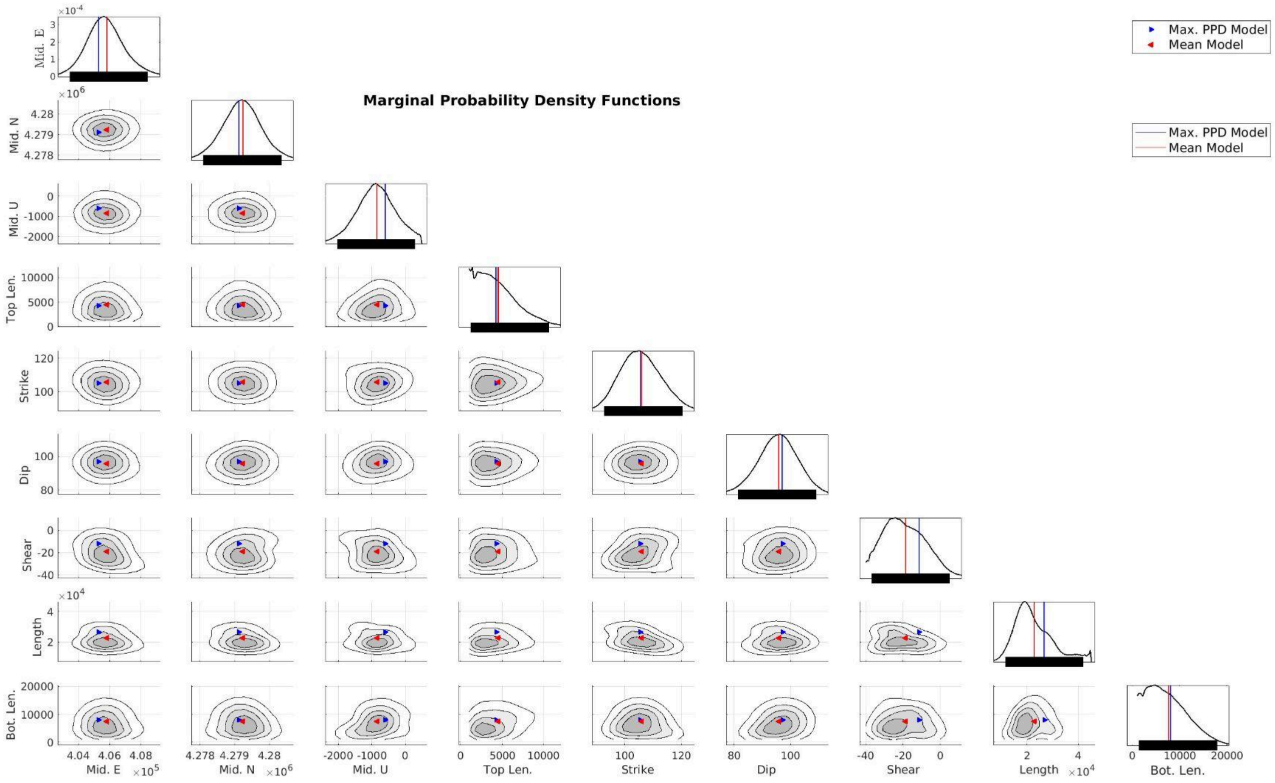

Figure S32. 1D Marginal probability density functions of the BEM model with vertical curvature [QDC]. The horizontal black bars represent the confidence limits from the inverted model solutions.

Text S2: Quantitative evaluation of a previously published conceptual model for the 2022 São Jorge seismic-volcanic unrest.

Recent work[7] proposes a conceptual model for the 2022 São Jorge unrest. Based on vertical GNSS displacements at station QEMD and the relocated seismicity pattern, this study interprets that (1) magma ascended a few days before the start of the seismic swarm, which was followed by (2) lateral dike propagation, and then (3) expansion of a lower-crustal sill-like body with an oblate ellipsoidal shape.

In component (1), a ~3 cm vertical uplift signal is observed ~two days before the seismic crisis, which reverses completely within 2–3 days, resulting in a net-zero displacement. To appraise this model, we analysed and compared the precise-point positioning (PPP) solution presented throughout this paper with a network-based solution independently processed using the GAMIT/GLOBK software (Fig. S2), which minimises common-mode noise effects. The PPP processing method computes individual solutions independently, and any correlation (common-mode errors) must be extensive, i.e., occurring at the same or larger scale than the Azores GNSS network. Additionally, any signal that is not coherent between these two solutions is likely a processing artefact. We find that neither the PPP nor network solutions robustly corroborate uplift at QEMD in the days preceding the seismic swarm. Although the PPP solution shows an apparent positive signal ~2 days before the start of the seismic crisis on 2022-03-19, this trend remains within the error bounds of the surrounding points (Fig. S2), and the network-based solution does not substantiate this signal. Moreover, we find that station, VLAZ, also on São Jorge, which Suarez et al.7 did not use in this study, does not show any precursory uplift signal either (Fig. S1).

Nevertheless, in addition to the GNSS observations, we conducted forward modelling to assess the robustness of a precursory inflation source. The ~3 cm uplift signal observed by Suarez et al.7 at station QEMD must have a different origin than the dyke, as it is not co-located with the InSAR deformation lobes, which are centred farther east of QEMD. Such a precursory uplift source must be located almost directly beneath QEMD, as it was recorded only in the vertical component and exhibited no significant horizontal displacements. To test this, we simulated GNSS displacement from a sill-like source located at the depth of the main seismicity clusters, capable of producing 4 cm uplift at QEMD (Figure S2.1). Remarkably, the simulated volume changes for such a sill are of the same order of magnitude as the volume intruded in the dike: 7.88×10^7 m³ for the planar quadrangle source and 6.56×10^7 m³ for the vertically curved quadrangle. However, station VLAZ recorded no significant horizontal or vertical displacements over the same period (Fig. S1). Given that the distance between VLAZ and QEMD is smaller than the estimated depth of the proposed source (8–12 km), a sill-like or spherical source affecting QEMD must also affect VLAZ. If the sill source were valid, VLAZ should have recorded ~1 cm horizontal displacement toward the west or northwest and ~1–2 cm uplift, neither of which is observed (Fig. S1). If a volcanic source drove the transient uplift, it would imply a rapid pressurisation (16-18 March) followed by equally rapid depressurisation on 19 March due to magma drainage into the dike. However, the lack of InSAR-detectable deformation in this area between 13 February and 27 March 2022 supports the interpretation that no net pressure change occurred.

Although we note that Suarez et al.[7] use a PPP solution with a common-mode bias (CMB) filter applied, we conclude that such a signal, if real, is neither robust nor substantial enough to be resolved or quantitatively modelled. Although volcanologically plausible, we suggest that some

coincident unmodeled atmospheric effect during the estimation of precise coordinates is a more likely cause for the ~3 cm transient uplift at QEMD reported by Suarez et al.[7]. Their Figure 4, Panel-n shows spurious uplift anomalies, with a similar order of magnitude, at QEMD much later in the seismic crisis, which they did not interpret as real signals, and could also indicate atmospheric noise.

Overall, compared to the dike component (2) in the conceptual model of Suarez et al.[7], our joint geodetic–seismic inversion results instead suggest a far broader and more vertically extensive dike intrusion, with no evidence for a Moho depth reservoir located at the locus of the seismicity. Nonetheless, we broadly agree with other aspects of their conceptual model, particularly the relocated seismicity, which shows remarkable consistency with our own results—underscoring the robustness of the seismic observations. We also agree that the far-field GNSS signals seem to have a longer, 4-5 day-long transient signal, but the amplitudes are too subtle to be reliably modelled. We acknowledge the importance of their observations and incorporate them into our broader interpretation, while highlighting key differences in data coverage and conceptual source geometry.

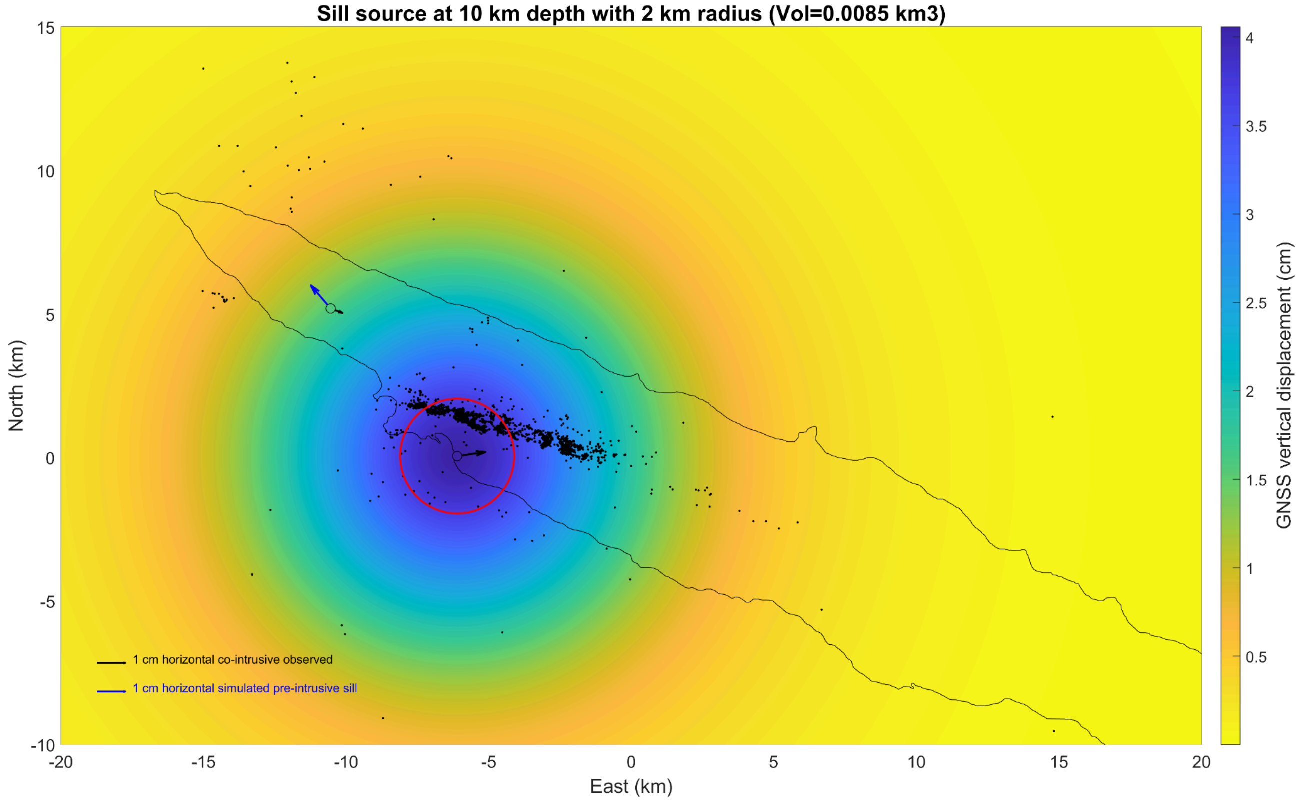

Figure S33 Simulated deformation field from a sill source located at 10 km depth with a 2 km radius, corresponding to a volume change of 0.0085 km³ ($8.5\times10^6 m^3$). The colour gradient represents the modelled vertical surface displacements, with cooler colours indicating greater uplift deformation. GNSS stations and seismicity are overlain for spatial reference.

References

1. Madeira, J., Brum da Silveira, A., Hipólito, A. & Carmo, R. Chapter 3 Active tectonics in the central and eastern Azores islands along the Eurasia–Nubia boundary: a review. *Geological Society, London, Memoirs* **44**, 15–32 (2015) https://doi.org/10.1144/M44.3.
2. Madeira, J. & Brum da Silveira, A. Active tectonics and first paleoseismological results in Faial, Picoand S. Jorge islands (Azores, Portugal). (2003).
3. Cao, A. & Gao, S. S. Temporal variation of seismic b-values beneath northeastern Japan island arc. *Geophysical Research Letters* **29**, 48-1-48–3 (2002) https://doi.org/10.1029/2001GL013775.
4. Ferreira, A. M. G., Marignier, A., Attanayake, J., Frietsch, M. & Berbellini, A. Crustal structure of the Azores Archipelago from Rayleigh wave ellipticity data. *Geophysical Journal International* **221**, 1232–1247 (2020) https://doi.org/10.1093/gji/ggaa076.
5. Matias, L. *et al.* The 9th of July 1998 Faial Island (Azores, North Atlantic) seismic sequence. *J Seismol* **11**, 275–298 (2007) https://doi.org/10.1007/s10950-007-9052-4.
6. Bagnardi, M. & Hooper, A. Inversion of Surface Deformation Data for Rapid Estimates of Source Parameters and Uncertainties: A Bayesian Approach. *Geochemistry, Geophysics, Geosystems* **19**, 2194–2211 (2018) https://doi.org/10.1029/2018GC007585.
7. Suarez, E. D. *et al.* São Jorge's Volcano-Tectonic Unrest in 2022: A Joint Interpretation Through GNSS and Fully Automated Seismic Analysis. *Pure Appl. Geophys.* (2024) https://doi.org/10.1007/s00024-024-03612-y.
