# Supplementary material for: Fault-mediated magma propagation and triggered seismicity revealed by the 2022 São Jorge Azores unrest
Source: Nat Commun. 2026 Apr 23;17:3531. doi: 10.1038/s41467-026-71668-6 (PMC13106810; doi:10.1038/s41467-026-71668-6)
Supplement: Supplementary file 2 — Description of Additional Supplementary File [file 41467_2026_71668_MOESM2_ESM.pdf]

Description of Additional Supplementary Files

Movie 1: 3-D animation showing the evolution of precursory seismicity, the geodetic model of the dike, and the spatial-temporal evolution of the main seismic swarm."
